# Plant Endemism Centres and Biodiversity Hotspots in Greece

**DOI:** 10.3390/biology10020072

**Published:** 2021-01-20

**Authors:** Konstantinos Kougioumoutzis, Ioannis P. Kokkoris, Maria Panitsa, Athanasios Kallimanis, Arne Strid, Panayotis Dimopoulos

**Affiliations:** 1 Division of Plant Biology, Laboratory of Botany, Department of Biology, University of Patras, 26504 Patras, Greece; ipkokkoris@upatras.gr (I.P.K.); mpanitsa@upatras.gr (M.P.); pdimopoulos@upatras.gr (P.D.); 2 Department of Ecology and Systematics, Faculty of Biology, National and Kapodistrian University of Athens, Panepistimiopolis, 15701 Athens, Greece; 3 Department of Ecology, School of Biology, Aristotle University of Thessaloniki, 54124 Thessaloniki, Greece; kalliman@bio.auth.gr; 4 Bakkevej 6, DK-5853 Ørbæk, Denmark; arne.strid@youmail.dk

**Keywords:** biodiversity conservation, CANAPE, conservation prioritization, ecosystem services, EU biodiversity strategy, GIS analysis, MAES indicators, Mediterranean flora, phylogenetic endemism, taxonomic diversity

## Abstract

**Simple Summary:**

Aiming to cope with the provisions of Aichi Biodiversity Targets, EU Biodiversity Strategy and EU Green Deal, we conducted the first nationwide, phylogenetically informed identification of vascular plant diversity hotspots and endemism centres in Greece. By this, we identified the most important factors that shaped them, and assessed the effectiveness of the Special Areas of Conservation of the Natura 2000 network in safeguarding them. Qualitative and quantitative results are provided and presented in thematic maps and relevant diagrams, highlighting areas of conservation importance, and identifying current protection scheme gaps. Simultaneously, our work contributes to national efforts for drafting Natura 2000 sites Management Plans, as well as to the MAES implementation in Greece.

**Abstract:**

Biodiversity hotspots (BH) cover a small fraction of the Earth’s surface, yet host numerous endemics. Human-induced biodiversity loss has been increasing worldwide, despite attempts to halt the extinction crisis. There is thus an urgent need to efficiently allocate the available conservation funds in an optimised conservation prioritization scheme. Identifying BH and endemism centres (EC) is therefore a valuable tool in conservation prioritization and planning. Even though Greece is one of the most plant species-rich European countries, few studies have dealt with the identification of BH or EC and none has ever incorporated phylogenetic information or extended to the national scale. Consequently, we are unaware of the extent that Special Areas of Conservation (SAC) of the Natura 2000 network efficiently protect Greek plant diversity. Here, we located for the first time at a national scale and in a phylogenetic framework, the areas serving as BH and EC, and assessed the effectiveness of the Greek SAC in safeguarding them. BH and EC are mainly located near mountainous areas, and in areas supposedly floristically impoverished, such as the central Aegean islands. A critical re-assessment of the Greek SAC might be needed to minimize the extinction risk of the Greek endemics, by focusing the conservation efforts also on the BH and EC that fall outside the established Greek SAC.

## 1. Introduction

Nearly 430,000 plant species occur on Earth [1]. Their distribution is uneven [2], due to historical and ecoevolutionary processes [3]. As a consequence, few areas or countries are mega-diverse in terms of overall plant species richness and few countries host more than 1000 endemic species [4,5]. These mega-diverse areas, which may include several countries or be part of a single country, experience intense anthropogenic pressure [5] and have been recognised as global biodiversity hotspots [6,7], since they exhibit exceptionally high plant species richness, high endemism levels and face very high levels of human-induced threat [7]. Climatically stable areas [8] may coincide with these global biodiversity hotspots, thus constituting macrorefugia [9] and more often than not, have high irreplaceability values (a measure of the conservation value of a given area [10,11]). Global biodiversity hotspots cover less than 20% of Earth’s surface, yet ca. 80% of all plant species are confined there [6,7], being thus extremely important in terms of conservation priority and even more so, in the Anthropocene era [12], which is characterised by elevated extinction rates [13,14,15]. In the last two centuries, species extinctions and biotic homogenization have been rapidly increasing [5,16], due to the synergistic effects of climate and land-use change [17,18,19] with evident spatiotemporal patterns [5], eventually leading also to an observable decline in ecosystem services. This homogenization and biodiversity deterioration trend is detected all over the globe [20,21,22], at all scales [18] and facets of biodiversity [23,24], despite plants’ innate resilience to extinction [25] (but see [26]).

In order to halt this trend, the Conservation on Biological Diversity set the Aichi Targets, among which the Targets 11 and 12 that aim to establish the minimum threshold of the percentage of terrestrial land under some form of protection and avert the extinction of known threatened species, respectively [27]. However, current conservation strategies have been inefficient regarding the prevention of biodiversity decline [28], since most conservation funds have been allocated in information gathering instead of recovery actions [29], but this inefficiency might also be a result of ecological time-lags [30]. Either way, the Aichi Targets might need to be re-evaluated when drawing the post-2020 biodiversity conservation agenda [31]. Due to the limited economic resources and the CBD’s ‘soft law’ approach [32], we are currently in urgent need to efficiently allocate the available conservation funds in an optimised and cost-effective conservation prioritization scheme [33,34,35]. These efforts should be accelerated [36,37] due to the intensifying negative effects of climate and land-use change on biodiversity world-wide [17,18,20,38,39]. In this regard, the identification of regional biodiversity hotspots (areas with elevated native and endemic species richness within global biodiversity hotspots [40]) and endemism centres (i.e., areas with significantly higher number of endemics than the surrounding landscape [41]) has been a useful and effective tool in conservation prioritization and planning [42,43,44]. However, the focus of conservation efforts may sometimes overlook such areas (e.g., [45]), so it is crucial that prioritization schemes are aimed at areas where the intersection between the different facets of biodiversity (i.e., taxonomic, phylogenetic and functional) is high [46] and at fine spatial scales [40], since endemism patterns are strongly scale dependent [41,47]. Spatial phylogenetics [48] via the incorporation of phylogenetic information (e.g., [49]), aid our understanding of the spatial biodiversity and endemism patterns and enable the effective design of conservation schemes [49,50,51,52,53,54], as well as elucidate the biogeographical origin of the area under study [46,50,55].

Five of the global biodiversity hotspots include islands or archipelagos [6,7], one of them being the Mediterranean Basin with its ca. 10,000 islands and islets. The latter constitutes the second largest hotspot in the world [56], with its largest islands exhibiting up to 18% plant endemism, which reaches up to 40% in their (sub-)alpine zones [57]. Several biogeographical and biodiversity studies have been undertaken in the Mediterranean Basin (e.g., [58,59,60,61,62,63,64,65]), yet few of them have dealt with the identification of phylogenetically-informed endemism centres and have been conducted at the subnational scale [22,66,67,68,69].

**Figure 1:**
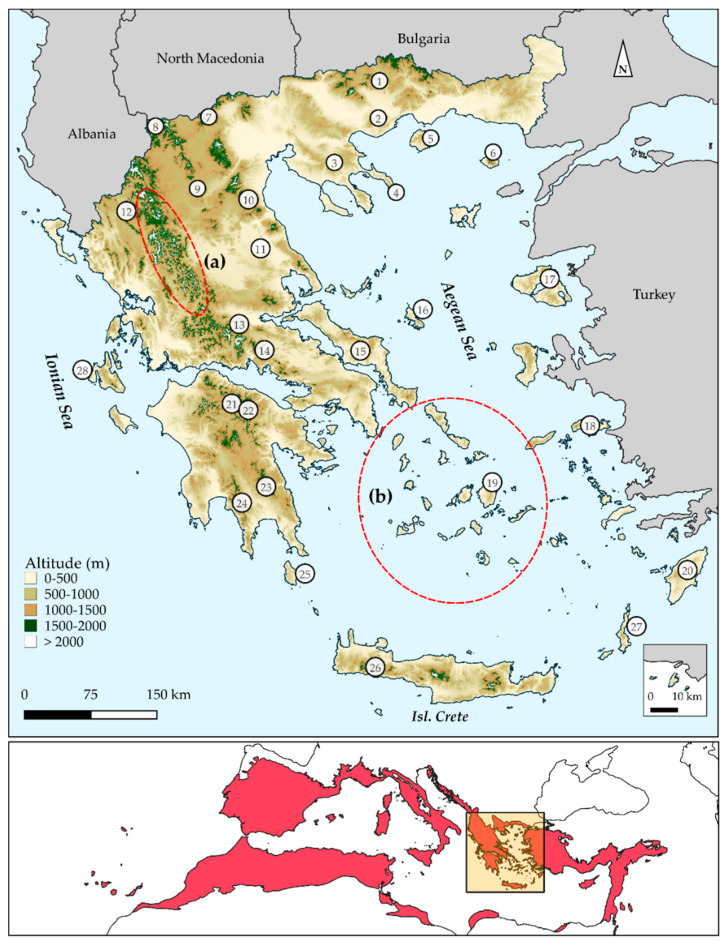
Upper panel: Map of Greece presenting major mountain massifs, as well as Aegean and Ionian islands that were identified as biodiversity hotspots and/or endemism centres and mentioned in the text. 1: Mt Falakron, 2: Mt Pangeon, 3: Chalkidiki mountains, 4: Mt Athos, 5: Isl. Thasos, 6: Isl. Samothraki, 7: Mt. Voras, 8: Lake Prespa area, 9: Mt. Vourinos, 10: Mt. Olympus, 11: Thessalian plain, 12: Vikos gorge, 13: Mt. Iti, 14: Mt Parnassos, 15: Isl Evvia, 16: Isl Skyros, 17: Isl Lesvos, 18: Isl Samos, 19: Isl Naxos, 20: Isl Rodos, 21: Mt Chelmos, 22: Mt Kyllini, 23: Mt Parnonas, 24: Mt Taygetos, 25: Isl Kithira, 26: Lefka Ori mountain range, 27: Isl Karpathos, 28: Isl Kefallinia. Red-dashed ellipses indicate (**a**) Pindos mountain range and (**b**) Kiklades island complex. Lower panel: Red colouring indicates the Mediterranean Biodiversity Hotspot (shapefile available from [130]).

Greece (Figure 1) is one of the most species-rich European countries [70], since more than 7000 native plant taxa occur there, with ca. 20% being endemic [70,71]. This high diversity and endemism are due to its topographical complexity, as Greece hosts ca. 8000 islands and islets and ca. 4800 mountain-tops [72,73], as well as to its long paleogeographical history [74,75,76,77]. Modern botanical exploration in Greece dates back to the 18th century [78,79] and a wealth of studies have been undertaken regarding the biogeography and the biodiversity patterns in Greece and its constituent archipelagos (i.e., the Aegean and the Ionian archipelagos [80,81,82,83,84,85,86,87,88,89,90,91,92,93,94,95,96,97,98,99,100,101,102,103,104,105,106,107,108,109,110,111,112,113,114]). Recent advances have addressed the Wallacean shortfall (i.e., the lack of knowledge on the geographical distribution of species) in Greece (e.g., [73,115,116,117,118,119,120]; Flora Hellenica Database (ongoing): ca. 1.2 M records). To date, very few studies have applied phylogenetic diversity metrics to macroecological analyses in Greece [22,103,121,122]. Equally few studies have dealt with the identification of regional biodiversity hotspots or endemism centres in Greece and they have been conducted at either a very coarse spatial scale or at the subnational level [22,116,123,124]. Furthermore, few studies exist regarding biodiversity conservation assessment and the efficiency of the protected areas network in Greece [125,126,127,128,129], yet none of them has incorporated any phylogenetic metrics in conservation prioritization analyses. Taking into consideration the ongoing progress on drafting species’ and habitats’ action plans and area-prioritization efforts inside Natura 2000 Special Areas of Conservation (SACs), the timing seems ideal for the first national, fine-scale and phylogenetically informed identification of biodiversity hotspots and endemism centres in Greece.

Our general goal is to improve the understanding of areas and patterns of endemism, biodiversity and biogeography based on a variety of spatially derived and phylogenetically informed metrics. More specifically, we aim to: (i) identify regional biodiversity hotspots and centres of endemism in Greece, (ii) investigate the factors leading to their creation and (iii) assess if and to what extent these areas are covered by SACs in Greece.

## 2. Materials and Methods

### 2.1. Environmental Data

Climatic data were obtained from the WorldClim [131] and the ENVIREM [132] databases at a ~5 km resolution. We extracted soil pH data from the SoilGrids database [133]. We extracted altitudinal and ruggedness data from the CGIAR-CSI database [134] and from [135], respectively. Since these data have a different resolution than the climatic data, we used functions from the ”raster” 2.6.7 R package [136] to aggregate and resample them in order for all environmental variables to have the same resolution. We then used functions from the “raster” 2.6.7 [136] and the ”spatialEco” 1.2-0 [137] R packages to estimate supplementary topographical variables (aspect, heat load index, slope, topographic position index and terrain ruggedness index) based on the altitudinal data. Geological data were derived from the 1:500,000 scale Geological Map of Greece [138] and classified as calcareous/not calcareous. As a measure of human impact, we used the Global Human Modification Index [139].

Following [140], we extracted paleoclimatic data from Paleoclim [141] and Oscillayers [142] and by using functions from the “climateStability” 0.1.1 R package [140], we were able to identify climatically stable areas in Greece (in terms of temperature, precipitation and their interaction-sensu [140]) for the past 4 My. We defined climate refugia as the 10% of cells (i.e., the 90% percentile) that had the highest score for the climate stability index.

From this initial set of 47 predictors, only thirteen (altitude, aspect, climate stability, heat load index, isothermality, potential evapotranspiration of the driest quarter, potential evapotranspiration of the wettest quarter, precipitation stability, slope, soil pH, the Thornthwaite aridity index and the topographic position index) did not have a collinearity problem (Spearman rank correlation < 0.7 and VIF < 10 [143]) and were thus included in our analyses. We used the “vifcor” function from the “usdm” 1.1.18 R package [144] to assess multicollinearity. All predictors were centred to a mean of zero and scaled to a standard deviation of one prior to the analysis [145], so as to enhance the comparability of parameter estimates in the subsequent analyses.

### 2.2. Species Occurrence Data

Greece hosts 7043 native plant taxa, 1435 of which are Greek endemics [70,71]. All subsequent analyses are based on the most extensive and detailed database of plants occurring in Greece [ca. 1.2 M occurrences—Flora Hellenica Database, Strid (continuously updated)]. All plant taxa were cross-checked for synonyms, following the nomenclature proposed by [70,71]. We used a grid cell resolution of ~5 km to match the resolution of the predictor variables, since preliminary tests indicated that this cell size delivered acceptable results considering the abrupt environmental gradients, high geodiversity and high endemic diversity characterising Greece. All subsequent analyses are based on the native and Greek endemic taxa that occur in Greece.

We calculated the number of native and Greek endemic taxa occurring in each grid cell (SR and ER, respectively). In order to put more emphasis on the range-restricted species (endemic or not), we also calculated the weighted endemism index (WE), as well as its corrected weighted variant (CWE [41,42,43]), using the functions provided from [146]. In WE, species are inversely weighted by their range size [147]. Consequently, CWE is the WE score divided by the SR (or ER) score. As a result, cells containing more range-restricted species will have a higher WE and/or CWE score than cells with fewer such species, thus providing crucial information regarding areas with exceptionally high diversity that can be regarded as biodiversity hotspots [42,43]. WE and CWE are thus range-weighted metrics and are consequently scale-dependent. We assessed the statistical significance of WE and CWE for both SR and ER by comparing the raw values of each grid cell to the 999 values of a null distribution, using the functions provided from [147]. As WE is usually strongly correlated with SR [41,148], we focused on CWE, which performs better in detecting biodiversity hotspots even when SR is not high [149]. We defined CWE, SR and ER diversity hotspots as the 1%, 5% and 10% of cells (i.e., the 99%, 95% and 90% percentile; L1, L2 and L3 diversity hotspots, respectively) that had the highest score for each of these indices. In a similar manner, we defined CWE diversity coldspots for the 1%, 5% and 10% percentile, using functions from the “phyloregion” [47,150,151] R package. Biodiversity hotspots are herein and hereafter defined as regional biodiversity hotspots (i.e., hotspots within global biodiversity hotspots [40]).

### 2.3. Phylogenetic Tree

A phylogenetic tree was generated following a “supertree” approach for all the taxa occurring in Greece, based on the phylogeny of seed plants by [152,153], using the largest dated mega-tree for vascular plants (GBOTB [153]). We appended any taxa present in Greece, but missing from the phylogeny, by adding them next to a randomly selected congener (except for the subspecies, which were added to the species they belong to), following [154] and [155], using the R code provided by [154] (https://github.com/oliverpurschke/sPlot_Phylogeny), as this procedure does not add any bias to subsequent analyses [154,155,156] and does not affect community-level phylogenetic metrics [157,158].

### 2.4. Biodiversity Analyses

We followed the categorical analyses of neo- and paleo-endemism (CANAPE) protocol for spatial phylogenetic analyses as set out in [48,159]. This procedure provides concrete insights regarding the evolutionary mechanisms that define biotas and enables solid and reliable conservation assessment and prioritization [160]. We carried out all the relevant analyses in Biodiverse version 3.0 [159] only for the Greek endemic plant taxa. We estimated phylogenetic endemism [161] and relative phylogenetic endemism [48], the core CANAPE metrics, and assessed their statistical significance using a null model in Biodiverse (i.e., the “rand_structured” option) as suggested by [48].

CANAPE characterizes grid cells into four different types of endemism centres: neo-, paleo-, mixed- and super-centres of endemism. Paleo- and neo-endemism centres have significantly high or low values respective to the relative phylogenetic endemism ratio (restricted long or short branches), respectively [48]. Mixed-endemism centres have a high percentage of both rare long and short branches, while super-endemism centres are a subdivision of mixed-endemism centres at the α = 0.1 level [48] (see Supplementary Materials for a thorough and in-depth explanation of the CANAPE protocol).

All analyses were performed using Perl wrapper functions to run Biodiverse in R modified from https://github.com/NunzioKnerr/biodiverse_pipeline.

### 2.5. Spatial Autoregressive Models

We used spatial autoregressive models with spatially autocorrelated errors (SAR) as outlined in [162], which take into consideration the spatial autocorrelation in parameter estimation [163], to investigate the relationships between the biodiversity indices we included in our analyses [i.e., number of native and endemic taxa (SR and ER, respectively), corrected weighted endemism of the native and the endemic taxa (CWE_NAT_ and CWE_END_, respectively), as well as phylogenetic endemism and relative phylogenetic endemism of the endemic taxa (PE and RPE, respectively)] with the uncorrelated predictors we included in our analyses. We used correlograms of the residuals of both SAR and generalized linear models to infer the degree of spatial autocorrelation [163], using functions from the “spdep” 1.1.3 R package [164]. The number of neighbours for SAR, as well as subsequent model selection was based on the lowest corrected Akaike information criterion (AICc [162]) value.

### 2.6. SACs Overlap

Traditionally, Priority Hotspots are defined based on the intersection of SR and CWE/WE hotspots [165]. We defined Priority Hotspots as any cells belonging to the top 1%, 5% and 10% of cells that had the highest score for both the CWE_END_ and PE indices, the two geographically-weighted variants of taxonomic and phylogenetic species richness, respectively. By doing so, we take into account two different facets of biodiversity, the geographically-weighted variants taxonomic and phylogenetic components of biodiversity, which more accurately identify biodiversity hotspots [42,43,48].

We overlapped the CWE_END_ hotspots, the areas identified as endemism centres, as well as the Priority Hotspots as previously defined with the Natura 2000 SACs (including SACs which are also Special Protection Areas, i.e., SAC/SPA) in Greece using R 4.0.3 and QGIS 3.14 in a Geographical Information Systems (GIS) analysis framework, in order to identify conservation gaps following [165]. SACs exclusively related to marine protection were excluded. Any cells identified as Priority Hotspots for both percentiles included in our analyses not covered by SACs (or their coverage was <10% [166]), were defined as Priority conservation gaps following [165].

## 3. Results

### 3.1. Biodiversity Hotspots and Centres of Endemism in Greece

**Figure 2:**
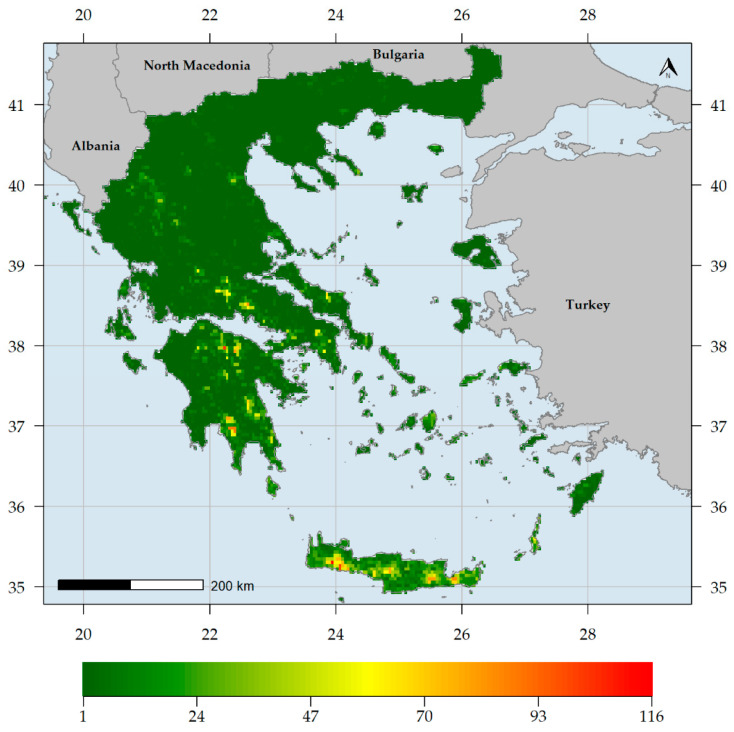
Endemic plant species richness in Greece (number of Greek endemic plant species per grid cell).

**Figure 3:**
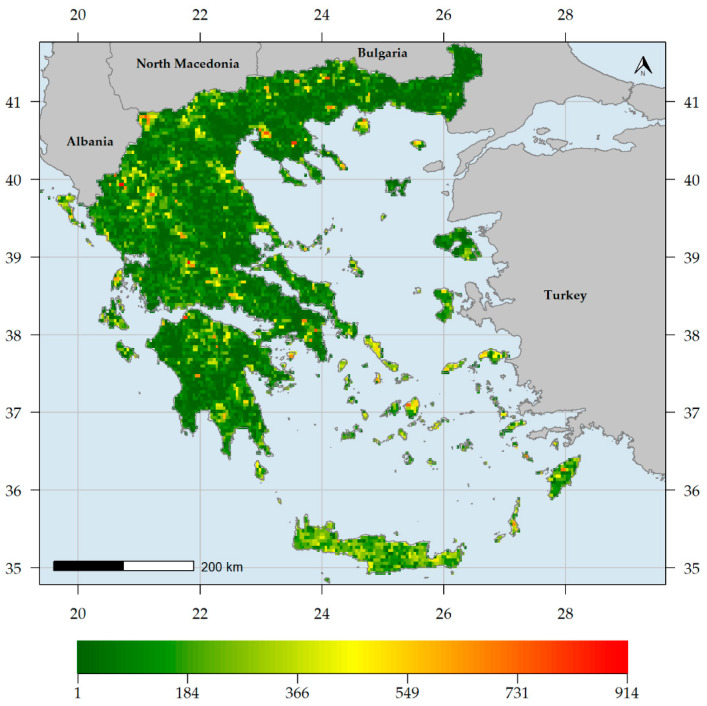
Native plant species richness in Greece (number of native plant species per grid cell).

Endemic species richness (ER) was higher in southern Greece, being highest in the Lefka Ori mountain range located in western Crete (Figure 2). Other mountain ranges in Crete, in northern and southern Peloponnese, as well as Mt. Parnassos in Sterea Ellas, exhibited high ER values (Figure 2). On the other hand, most of mainland Greece and the Ionian islands had very low ER values (Figure 2). Native species richness (SR) was higher in northern Greece, being highest in Mt. Timfristos in Sterea Ellas (Figure 3). Other areas with high SR values are mainly found in northern Greece, such as the Vikos gorge (in the N Pindos mountain range), the wider Lake Prespa area, Mts. Cholomon and Chortiatis in Chalkidiki peninsula, Mt. Falakron in NE Greece, as well as the central Aegean island of Naxos (Figure 3). The central and southern Aegean islands had higher SR values than most low-elevation mainland areas and the Ionian islands (Figure 3).

**Figure 4:**
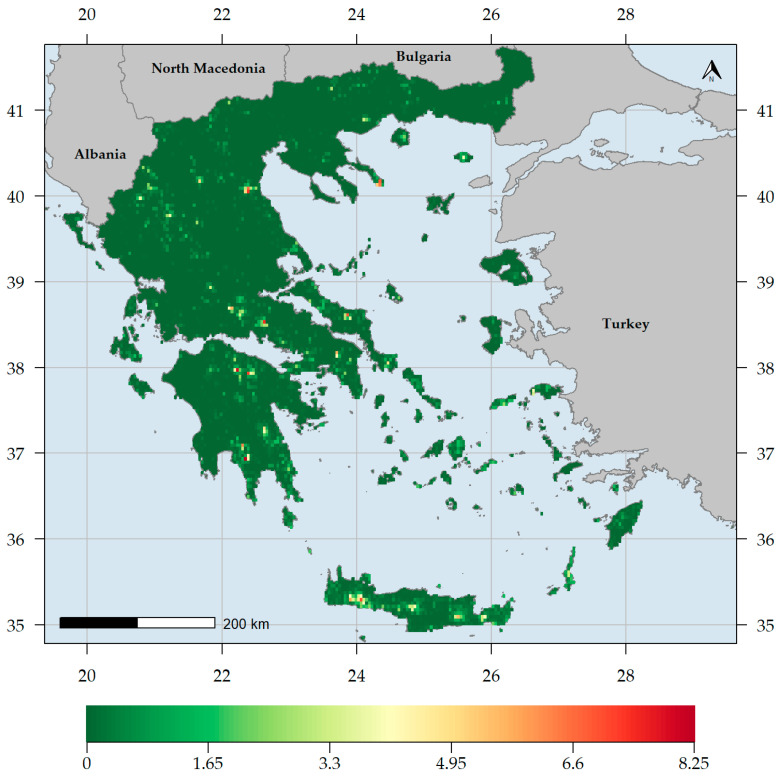
Corrected weighted endemism values for the Greek endemic plant taxa (CWE_END_) occurring in each grid cell in Greece.

**Figure 5:**
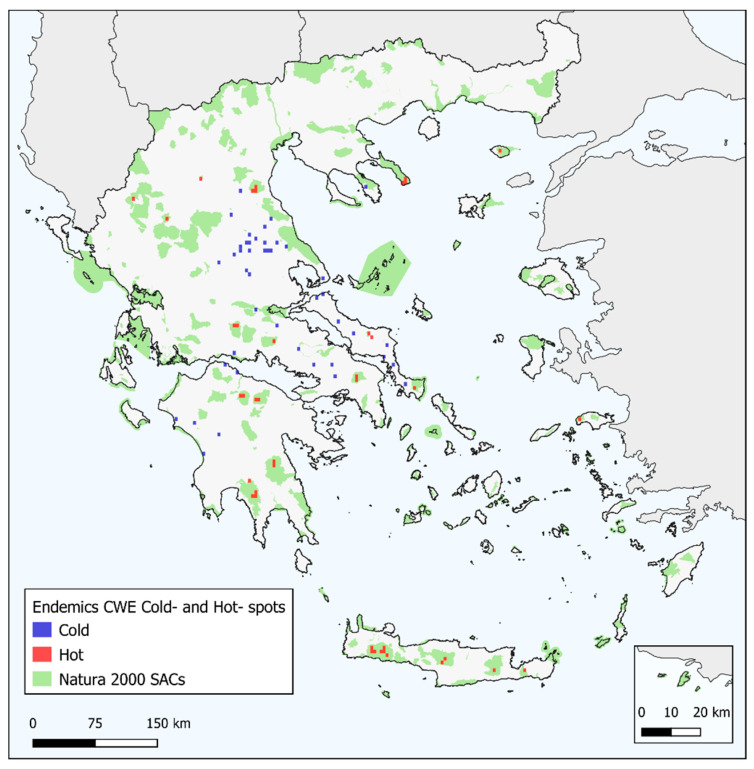
Red colouring indicates cells with Level-1 (top-1%) CWE_END_ values. Blue colouring indicates cells with values from the 1% percentile (CWE_END_ coldspots). Green polygons depict the Natura 2000 SACs in Greece.

**Figure 6:**
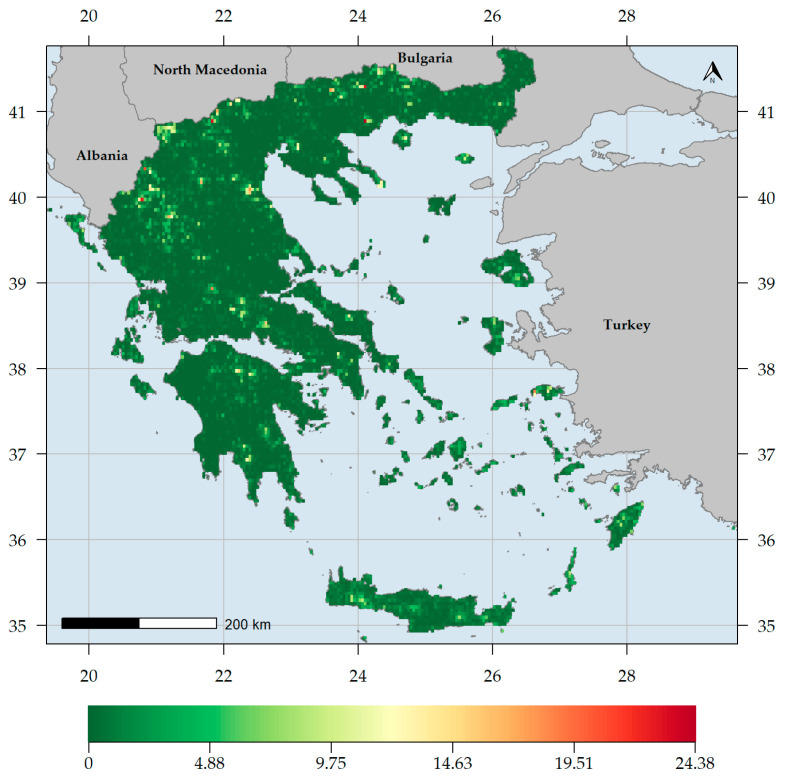
Corrected weighted endemism values for the native plant taxa (CWE_NAT_) occurring in each grid cell in Greece.

CWE values for the endemic taxa (CWE_END_) ranged between 0 and 8.25, the highest value found on Mt. Taygetos in southern Peloponnese (Figure 4). Most L1-L3 CWE_END_ hotspots occurred in high-elevation areas in Crete and the Peloponnese (Figure 5, Figures S1 and S2). Most CWE_END_ coldspots occurred in mainland Greece near the Thessalian plain, in Evvia and western Peloponnese regarding L1 CWE_END_ coldspots (Figure 5) and central and northern Greece in general, regarding L2-L3 CWE_END_ coldspots (Figures S1 and S2). Important L1 CWE_END_ hotspots occurred on Mts. Oiti and Parnassos, on Mts. Olympus and Athos, on Mt. Vourinos [the only L1 CWE_END_ hotspot on ultramafic rocks (i.e., with silica content below 45% and a high concentration of heavy metals [167])], as well as Samos and Samothraki (Figure 5). Several Aegean islands (Amorgos, Antikythera, Astypalaea, Crete, Folegandros, Ikaria, Karpathos, Kythera, Lesvos, Naxos, Rodos, Samos, Samothraki, Skyros, Symi and Thasos) and only one Ionian island (Kephallinia) emerged as L2-L3 CWE_END_ hotspots (Figures S1 and S2). Most L1 CWE_END_ hotspots had significantly high values according to the randomization tests (Figure S3). CWE values for the native taxa (CWE_NAT_) ranged between 0 and 24.38, the highest value found on Mt. Pangeon in NE Greece (Figure 6), followed by Mts. Falakron and Voras (Kajmakcalan) in NE Greece and Vikos gorge in Epirus. Every high CWE_NAT_ area had significantly high values according to the randomization tests (Figure S4), while some central and northern Aegean islands, as well as Crete, had significantly low values according to the randomization tests (Figure S4).

**Figure 7:**
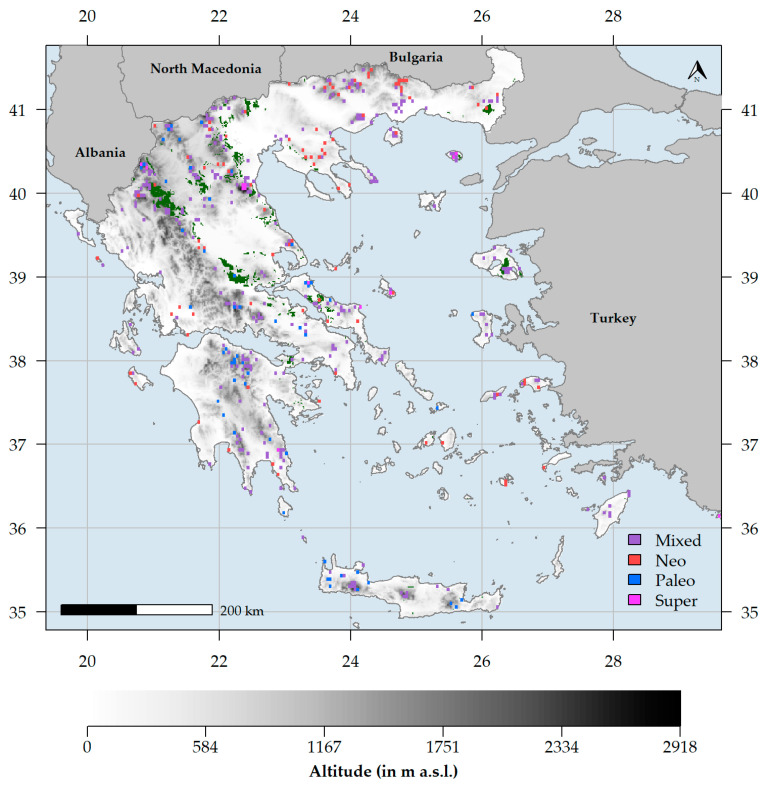
Map of significant phylogenetic endemism (PE) identified by the categorical analysis of neo- and paleo-endemism (CANAPE) analysis for the endemic plant taxa occurring in Greece. Dark green colouring indicates the areas where ultramafic rocks occur in Greece.

**Figure 8:**
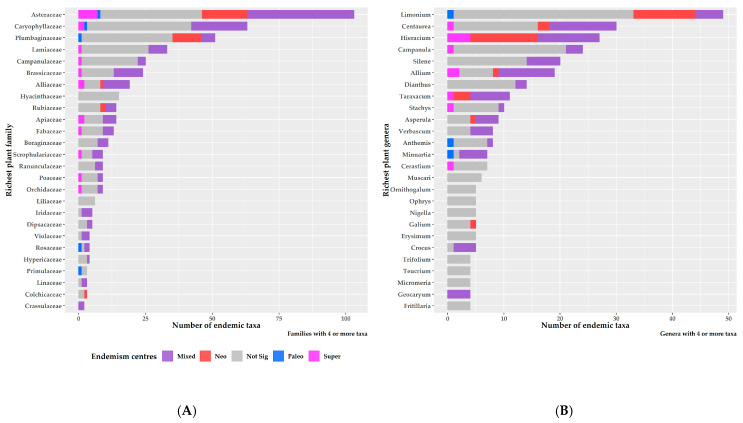
Richest plant families and genera [left (**A**) and right (**B**) panel, respectively] exclusively occurring in a single endemism centre type.

The endemism centres were mainly concentrated within and at the periphery of the Greek mountain massifs (Figure 7). Areas of mixed-endemism were the most common, followed by neo-, paleo- and super-endemism areas (301, 89, 54 and 13, respectively; Figure 7). Excluding the insignificant endemism centres, mixed-endemism areas were the most species-rich, while paleo-endemism areas were the poorest in terms of species richness (Table S1). At the family level, Asteraceae had the most species occurring in a single endemism centre type, followed by Caryophyllaceae and Plumbaginaceae (Figure 8A). At the genus level, *Limonium* had the most species occurring in a single endemism centre type, followed by *Centaurea*, *Hieracium* and *Campanula* (Figure 8B). Most neo-endemics belong to Asteraceae and Plumbaginaceae (Figure 8A), with *Hieracium* and *Limonium* being the most species-rich in neo-endemics (Figure 8B).

### 3.2. Characteristics of Endemism Centres

**Figure 9:**
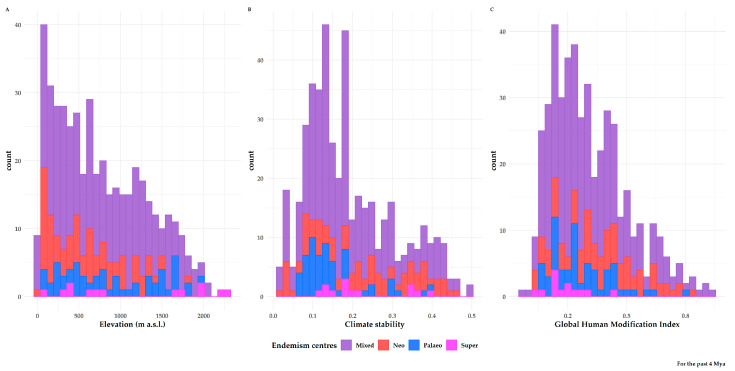
From left to right: (**A**) Altitudinal distribution, (**B**) Climate stability values and (**C**) Global Human Modification index values of the different types of endemism centres identified by the CANAPE analysis in Greece.

Centres of neo-endemism occurred at a significantly lower altitude than all other types of endemism centres (Kruskal–Wallis ANOVA: H = 4000, df = 4000, p = 0.2; Table S2; Figure 9A) and in climatically-stable areas (Figure S5). Paleo-endemism centres had significantly lower climate stability values than all other types of endemism centres (Kruskal–Wallis ANOVA: H = 4000, df = 4000, *p* < 0.1; Table S2; Figure 9B). Regarding human impact, neo- and super-endemism centres experienced the highest and lowest impacts of human disturbance, respectively (Kruskal–Wallis ANOVA: H = 4000, df = 4000, *p* = 0.5; Table S2; Figure 9C). Finally, paleo-endemism areas occurred in southern and western Greece compared to neo-endemism areas, which occurred in northern and eastern Greece (Kruskal–Wallis ANOVA: H = 400, df = 200, *p* < 0.01; Table S2; Figure 7).

### 3.3. Factors Shaping Biodiversity Hotspots and Endemism Centres

Elevation was the most important predictor of all the biodiversity indices we included in our analyses, except for phylogenetic endemism (PE), with Nagelkerke pseudo-R-squared (GR^2^) ranging between 18.1% and 48.0% (Table 1). Climate-related variables (e.g., aridity, evapotranspiration of the driest quarter, climate stability) usually followed elevation in variable significance for SR, ER, CWE_END_, CWE_NAT_ and RPE (Table 1). Regarding PE, evapotranspiration of the driest quarter was the most important variable, followed by the occurrence of calcareous rocks (Table 1).

### 3.4. Overlap with SACs

**Figure 10:**
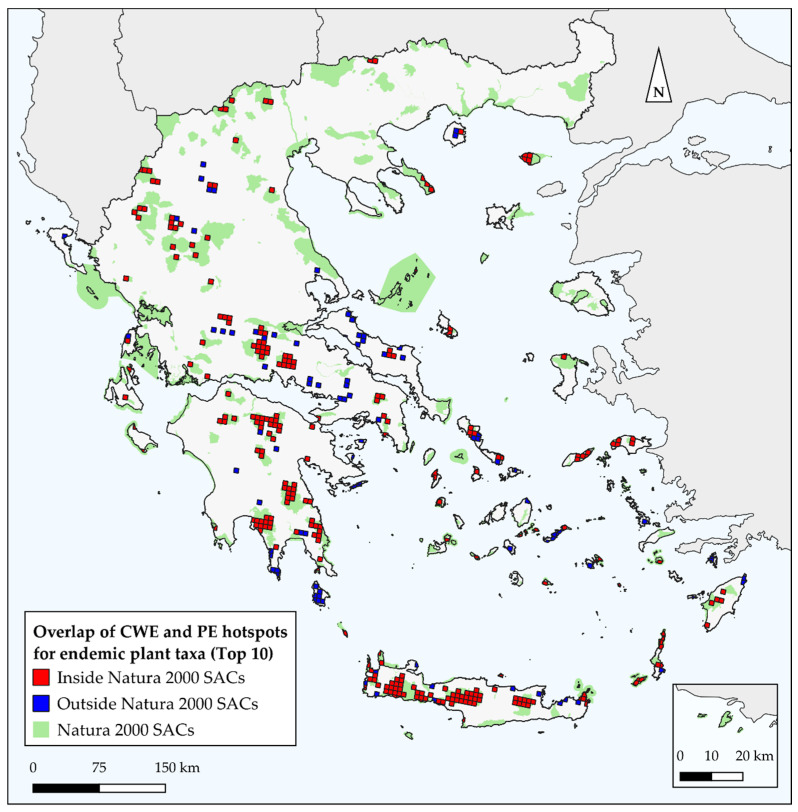
Red colouring indicates cells identified as Priority Hotspots with Level-3 (top 10%) CWE_END_ and PE values that are inside the Natura 2000 SACs. Blue colouring indicates cells identified as Priority Hotspots with Level-3 (top 10%) CWE_END_ and PE values that are outside the Natura 2000 SACs. Green polygons depict the Natura 2000 SACs in Greece.

The overlap between the SACs of Greece and endemism centres detected by CANAPE was rather high and ranged between 38.9% and 84.5% (when excluding the insignificant endemism centres; Table S3; Figure S6). The overlap between the SACs in Greece and the CWE_END_ hotspots was in general higher than that reported for endemism centres and ranged between 72.2% and 95.7%, depending on the percentile threshold used to define L1-L3 hotspots (Table S5; Figure 5). The same trend was observed for the overlap between SACs in Greece and the areas recognized as Priority hotspots (Table S4; Figure 10, Figures S7 and S8). The conservation gaps in Greece thus range from 3.3% to 61.1% (Tables S3 and S4).

## 4. Discussion

The Mediterranean Basin is one the richest and largest biodiversity hotspots in the world, due to its rugged topography and its intricate geography and orography since the Palaeocene [168]. The Iberian, Italian and Balkan peninsulas have largely shaped the region’s biogeographical patterns [169,170], as a result of complex interactions between geographical, topographical and climatic factors [170,171,172,173,174,175], which led to the occurrence of numerous refugia that fostered both the persistence, as well as the diversification of several species [176,177,178,179]. More specifically, the Balkan Peninsula due to its patchy and mountainous landscape, has provided shelter to several cold- and warm-adapted species [180,181,182] of usually relict origin [173], while allowing a westward migration from Asia to Europe during climatic oscillations and in-situ speciation [183,184,185,186,187], thus eventually leading to its currently observed high endemism rates [188,189,190]. Greece stands out as one of the most diverse Mediterranean and European countries in terms of plant species richness and has a rich and long floristic exploration record, with many of its northern mountain massifs probably acting as refugia [70]. Here, we located for the first time at a national scale, the areas serving as biodiversity hotspots, as well as the areas that act as diversity cradles and museums (centres of neo- and paleo-endemism, respectively), identified the most important factors that shaped them, and assessed the effectiveness of the Special Areas of Conservation of the Natura 2000 network in safeguarding these areas. By doing so, we moved one step towards the completion of Aichi Biodiversity Target 11 in Greece.

### 4.1. Biodiversity Hotspots in Greece

A large number of Greek mountains, as well as some Aegean islands are among the main Mediterranean and Balkan biodiversity hotspots [191] and are rich in endemics [123,190,192]. This is more pronounced in the southern Greek mainland and Crete: the Peloponnese hosts the most Greek endemic species [70,71,89,193], while Crete constitutes the hottest endemic island hotspot in the Mediterranean [57]. Islands and island regions in general were thought to be poorer in terms of species richness compared to the Greek mainland, due to the area-effect and the existence of a more ‘balanced’ continental flora [111,194,195]. More specifically, the central Aegean islands were considered as having an impoverished flora due to the ”Kykladenfenster” phenomenon (i.e., the absence of certain plant taxa from the central Aegean islands that are present in mainland Greece and Asia Minor) [195] and even though there are cracks to this view [92,111,112,114], it has not yet been established [115]. Overall, endemism rate seems to decline in a NW-SE axis, with a small fraction of narrow endemics occurring above 1500 m a.s.l. [193,196,197] and elevation-driven isolation has played a significant role in shaping the patterns of endemic species richness in southern mainland and insular Greece [22,89,90,91,94].

Generally, higher and drier areas host more native and endemic plant taxa in Greece (Table S3, Figure 2, Figure 3, Figure 4 and Figure 6), with native and endemic species richness being higher in northern and southern Greece, respectively (Figure 2 and Figure 3). Elevation emerged as the most important predictor for all the taxonomic diversity metrics (unweighted or weighted; ER/CWE_END_ and SR/CWE_NAT_) spatial patterns in Greece, and several mountain ranges materialised as the main (native or endemic) diversity centres in Greece. This pattern has been observed at the local scale in Greece [22,84,90,91,93,114] and at the regional scale, in areas such as the Iberian Peninsula [198,199], Mexico [200], New Zealand [201] and Iran [202], as well as at the global scale [203]. Increased topographical complexity, along with stable climatic conditions during the Quaternary stadials and interstadials, may have triggered ecological speciation, while reducing extinction risk and gene flow, thus promoting allopatric speciation and in the same time allowing the persistence of warm-adapted species [191,202,203,204,205,206,207]. According to the Mountain-Geobiodiversity-Hypothesis (MGH [208]), mountain formation and uplift determine regional biodiversity patterns by providing refugia and an increased chance of allopatric speciation if in a given mountain massif: (i) steep environmental gradients along its elevational range exist, (ii) Quaternary climatic oscillations that aided the ‘species-pump’ effect were recorded and (iii) its terrain is highly rugged. During more favourable conditions (e.g., during the interstadials) plant species would have tracked their niche [209,210] and based on the possibility of connectivity among sites on a given mountain massif, diversification might have increased (Flickering Connectivity Hypothesis [211]). This seems to be the case in Greece as well, since several mountain ranges were identified as biodiversity hotspots, the most prominent of which being the Lefka Ori mountain range in Crete regarding the endemic plant taxa (Figure 4) and Mt. Pangeon in NE Greece regarding the native plant taxa (Figure 6). Many Greek mountain massifs, such as the Pindos mountain range, are included among the most important Mediterranean glacial refugia [182] and in this context, Mt. Pangeon has acted as a local refugium for some relict (microthermic) species [212], while other mountainous areas in central Greece served as refugia for Tertiary relicts [70,184], for primitive Caryophyllaceae taxa [213] and for xerothermic species [214,215]; the latter due to the occurrence of ultramafic rocks [98,123,216]. One such mountain is Mt. Vourinos, a serpentine “island” in the limestone-dominated valley of Thessalia [123], which is among the areas with statistically significant higher CWE_NAT_ values and constitutes the only L1 CWE_END_ hotspot on ultramafic rocks (Figure 5); all other L1 CWE_END_ hotspots occur on calcareous substrates, since Greek endemics seem to be in most cases habitat specialists, preferably occurring on limestone cliffs [70,199], probably due to human-induced pressure [217,218].

The southern Greek mainland, as well as Crete, were thought to be regional endemic hotspots, while the rate of endemism in northern Greek mainland was considered to be significantly lower compared to the Peloponnese and the southern Aegean islands [70,71,90,193,219]. Mt. Taygetos, the highest and one of the most topographically complex Peloponnesian mountains, abides to this notion, since it emerged as the most important CWE_END_ area in Greece, probably as a result of the peninsular effect and of ecological isolation [89,213,219]. This seems superficially in harmony with what has been perceived as the norm regarding the endemism patterns in Greece [193]. Our findings, however, paint a slightly different picture, as several northern mainland mountains, such as Parnassos, Oiti, Olympus and Athos are identified as L1 CWE_END_ hotspots (Figure 5), along with the Cretan and the Peloponnesian mountain massifs. This phenomenon becomes more evident, when considering L2 and L3 CWE_END_ hotspots (Figures S1 and S2), as most of these hotspots occur in central and northern mainland Greece. This may be linked to the MGH and FCH hypotheses [208,211,220], as well as to the fact that most of these mountains have acted as Quaternary refugia for many relict species [70,182,184] and as diversity cradles probably due to numerous hybridization events (e.g., as in the genus *Hieracium* [221,222,223]) and elevation-driven ecological isolation [203,223,224]. Several Aegean islands are also identified as L1 CWE_END_ hotspots, confirming previous studies stating that some of the central and eastern Aegean islands are important Greek endemic hotspots [84,88,114], while also lending weight to the rebuttal of the impoverishment of the central Aegean flora [195]. Even when taking into consideration the native flora, most central Aegean islands have higher native species richness than most lowland mainland areas (Figure 3) and only Andros and Syros seem to have statistically significant lower CWE_NAT_ values (Figure S4). These incongruences might be partly due to the different methodologies used, in addition to the quality of the data at hand: we used the most extensive occurrence database to date regarding the native plant taxa occurring in Greece and the CWE metric that puts more emphasis on range-restricted species, thus being more reliable in detecting biodiversity hotspots [41,42,43].

### 4.2. Endemism Centres in Greece

Mountains may promote or hinder species dispersal depending on the ease of connectivity [211] and by doing so, they can increase the diversification rate either by reducing gene-flow or by providing the ground for intense hybridization and polyploidization events [220,225]. Even though mountain radiations mainly occurred during the Pleistocene [226,227], some may have occurred during the Pliocene [220]. Balkan Mountains constitute the main Pleistocenic refugia for several Tertiary relics [228,229], are regarded as genetic diversification havens [176,178,186] and seem to have shaped the diversification of several species complexes [187,191]. The Greek mountains are no exception to this rule [230], as they host numerous Tertiary relicts [70,182,184], providing shelter either to cold- or warm-adapted species during the climatic oscillations of the Pleistocene [214,215,231] and have given rise to several narrow endemics [191,219]. Our results corroborate this hypothesis, since in Greece, the species-rich, mixed-endemism centres dominate (Table S1; Figure 7 and Figure 9) and most endemism centres occur in or near montane regions (Figure 7 and Figure 9), suggesting that the Greek mountains act both as diversity cradles and museums—A trend observed elsewhere as well [51,53,200,232,233]. Several mixed- and paleo-endemism centres occur in the N and S Pindos mountain range in northern and central Greece (Figure 7), thus confirming the hypothesis that it constitutes a major endemism centre and phylogeographical hotspot in the Mediterranean Basin [182]. More specifically, Mts. Parnassos and Oiti in central Greece have a high concentration of mixed-endemism centres (Figure 7), probably a result of their geographical setting and topographical heterogeneity, serving as stepping stones between two major Mediterranean diversity hotspots, namely the Peloponnese and the Pindos mountain chain [190]. Mt. Olympus, the highest mountain in Greece, also forms an important endemism centre (Figure 7), as several local endemics and Tertiary relics occur there, such as *Jankaea heldreichii* [70,234].

Paleo-endemism centres tend to occur in lower latitudes, higher altitudes and in less climatically stable areas in Greece than neo-endemism centres (Table S2; Figure 7 and Figure 9). This might be due to elevation-driven ecological isolation [90,207,223], the increased ruggedness of the southern mainland and insular Greek mountains [90,219], their long-lasting geographical isolation and the existence of Pliocenic islands that now constitute large mountain chains (as in the case of Crete [22]) that may have reduced gene-flow, thus enabling the persistence of those paleo-endemic species, a phenomenon observed elsewhere as well [233]. On the other hand, neo-endemism centres occur in sites of greater climatic stability and intense human pressure (Table S2; Figure 7, Figure 9 and Figure S5) and are mainly found in NE lowland Greece and to a lesser degree, in southern Peloponnese, Evvia and in other few Aegean islands (Figure 7). Our results align with the hypothesis that low climate-change velocity areas may be linked with the existence of neo-endemism centres [235,236], since long-term climate refugia promote speciation [236,237,238]. Another important factor in shaping neo-endemism centres in Mediterranean-type ecosystems is aridification and dry climate conditions in general [68,199,239,240], as the intensification of summer drought since the Pliocene, resulted in increased spatial and genetic isolation [59,168,184,210] and subsequent accelerated speciation of numerous species complexes in the Mediterranean [53,241,242,243]. This seems to be the case in Greece as well, since aridity and dry climatic conditions in general (Table 1) drive RPE patterns and may explain the existence of several neo-endemism centres in areas experiencing such conditions, viz. the lowlands in NE Greece and the coasts of some central Aegean islands (i.e., Paros, Naxos and Astypalaea—Figure 7), where recent radiations occurred for many species complexes, such as *Limonium* [242], *Nigella* [244,245,246] or *Hieracium* [221,222] spp. The southern Peloponnesian lowlands also contain neo-endemism centres, and this might be ascribed to their older geological age and their elevated ecological isolation due to the sea-barrier existing in their south and the large mountain massifs that lie to their north [89,219]. The neo-endemism centres that lie in Evvia (Figure 7) may be linked to the vast outcrops of ultramafic rocks, which have largely driven the evolution of many plant lineages [98,122,213,214].

### 4.3. Conservation Prioritization–Management Implications

A central aim of conservation biology is the identification of biodiversity hotspots, i.e., areas with a relative high species richness and endemism, which pinpoint underlying ecological and evolutionary patters and processes [247], at global and regional scales, with the latter termed ”hotspots-within-hotspots” [40]. This process enables the efficient and cost-effective conservation management of biodiversity by deftly allocating the limited available resources (e.g., [248]). The identification of biodiversity hotspots relies at its core on species-level metrics, such as species richness (e.g., endemic and threatened species richness) [7,249,250], so that the identification of endemism centres is crucial for regional conservation planning.

Most Greek endemic plant taxa have a narrow distribution and are considered threatened [195,251]. The established Greek SACs cover up to ca. 28% of the country’s area [252] and seem to adequately protect the endemism and diversity centres, when considering CWE_END_ values (Table S3; Figure 5). In this regard, the conservation gap in Greece might seem minimal. However, it is pivotal for prioritization schemes to search for areas where there is high intersection between different facets of biodiversity (e.g., taxonomic and phylogenetic) [46] and thus relying only on one of these facets [165], may be misleading regarding the protection and conservation status of the areas identified as biodiversity hotspots. By using the Priority Hotspots metric as defined herein, we were able to overcome this predicament. Our results suggest that the conservation gap in Greece is actually not minimal, as suggested by the CWE metric, but instead it is quite significant (Table S4; Figures S6 and S7), at least for the L2-L3 Priority Hotspots. Despite the fact that the overlap between the SACs and the Priority Hotspots identified in our study meets the 10% threshold, which refers to the minimal percentage of a range that must be overlapped by the SACs in order for the species to be considered covered [253,254], as well as meeting the Aichi Biodiversity Target 11 threshold for important biodiversity areas to be effectively conserved and equitably managed via ecologically representative and well-connected systems of protected areas, such as SACs, a significant amount lies outside any protection scheme. The same trend is observed for the endemism centres as well (Table S3; Figure S6). Moreover, these thresholds should be always considered when referring to species with a narrow geographic range, such as the Greek endemics (or range-restricted taxa and local endemics). Therefore, a more careful assessment of the current SACs extent is recommended, in order to protect the Greek endemics as they are integral structural and functional elements of habitat types. As anthropogenic pressure will increase in the coming decades due to climate- and land-use change [17,18,19], even for Type-III organisms such as orchids [255], a critical re-assessment of the current SACs might indeed be needed in order to minimize the extinction risk of the Greek endemics, by focusing the conservation efforts also on the Priority Hotspots that fall outside of the established Greek SACs or on mixed-endemism centres. The former host many rare species [42,43], while simultaneously representing evolutionary history and, potentially, adaptive capacity [247]. The latter on the other hand, contain a large number of both neo- and paleo-endemic species. Thus, by aiming the available human and economic resources in the protection and management of either of these areas would be socioeconomically cost-effective and align with the ongoing efforts on expanding SACs to capture and protect the phylogenetic aspect of biodiversity and evolutionary heritage in general [256,257].

Regarding the importance of this work in current conservation, management and policy efforts, our results contribute to the implementation of the ongoing state efforts via drafting Special Environmental Studies (SES) for the Natura 2000 Network sites in Greece, as well as to the Life Integrated Project with the acronym “LIFE-IP 4 Natura” [258], led by the Hellenic Ministry of Environment and Energy. More specifically, the identified biodiversity hotspots, endemism centres and Priority Hotspots are fundamental for the aforementioned, nationally designed, holistic approaches of conservation management and sustainable development since they: (a) provide spatial explicit biodiversity information which can be incorporated in the creation of protection zones via the SES implementation and (b) fulfil the adopted biodiversity indicators for the MAES (Mapping and Assessment of Ecosystem and their services) implementation in Greece (i.e., the indicators of Floristic diversity and Endemic diversity—[252]). They do so, as they directly correspond to the project’s needs, while simultaneously meeting all index requirements as proposed by [259] (i.e., being scientifically sound, supporting environmental legislation, being policy-relevant, including habitat and species conservation status, including soil-related information, being applicable for natural capital accounts, being spatially explicit, supporting baseline and being sensitive to change). Our work thus acts not only as a dissemination medium of scientific results and outcomes, but also provides the robust baseline for future assessments, where data is available at any scale (local to national). For instance, a similar analysis could be conducted for certain selected taxa of particular economic and/or cultural importance and not only for conservation management. The endemic Lamiaceae taxa, with documented importance regarding their properties (e.g., aromatic, medical, antimicrobial, traditional medicine, environmental interest) [260], is a characteristic group of economic and cultural importance and could be used for a similar case-study assessment. Moreover, our results cope with the needs of the National and EU Biodiversity Strategy, as well as with the proposals made by the EU Green Deal, which urge Member States to follow, in order to bend the curve of biodiversity loss, ecosystem melioration at all scales and the subsequent provided social equity and well-being.

## 5. Conclusions

Spatial phylogenetic analyses provided informative aspects of biodiversity related to evolutionary history, distinctiveness, and uniqueness. This study presents for the first time a nationwide, phylogenetically informed identification of vascular plant diversity hotspots and endemism centres in Greece. These areas are mainly located near or at mountain-tops and mountain ranges, as well as in areas which were until now thought of as floristically impoverished or were in one way or another, overlooked. By applying a metric which takes into account the geographically-weighted variants of both taxonomic and phylogenetic diversity, we were able to discern the conservation gaps in the Greek Natura 2000 network, regarding the areas that serve as both taxonomic and phylogenetic hotspots, providing evidence that the incorporation of phylogenetic metrics in national conservation strategies does indeed unveil patterns yet unseen. Owing to the detailed data availability used for this study, our outcomes provide results adequate for decision making and policy support, for the implementation of the National Biodiversity Strategy as well as to support the requirements of the European Green Deal.

## Figures and Tables

**Table 1:** Best spatial autoregressive error models (SAR_err_) for the relationships among the number of native (SR) and endemic (ER) taxa, corrected weighted endemism of the native (CWE_NAT_) and the endemic taxa (CWE_END_), phylogenetic endemism (PE), relative phylogenetic endemism (RPE) and the predictor variables. GR^2^: Gelkerke pseudo-R-squared. AICc: Akaike Information criterion corrected for small samples. Asterisks denote: * *p* < 0.05, ** *p* < 0.001. AIT: Thornthwaite’s aridity index. CS: Climate stability. Iso: Isothermality. PET_DRQ_: potential evapotranspiration of the driest quarter. PET_WETQ_: potential evapotranspiration of the wettest quarter. TPI: topographic position index. Geology refers to whether a grid cell is classified as calcareous or not. Only the statistically significant variables are shown.

Response	Predictor	Coefficients	GR^2^	AICc
**SR**	AIT	11.4 **	18.1	83671
	TPI	−43.02 **		
	PET_DRQ_	−7.64 **		
	PET_WETQ_	8.25 **		
	pH	10.17 **		
	Slope	20.66 **		
	Altitude	43.37 **		
	Iso	−19.78 **		
**ER**	AIT	1.73 **	48.0	44316
	PET_DRQ_	−0.97 **		
	pH	0.71 **		
	Slope	0.73 **		
	Altitude	4.64 **		
	Iso	−0.91 **		
	CS	−1.52 **		
**CWE_NAT_**	AIT	−0.14 **	28.5	24798
	TPI	−0.18 **		
	PET_DRQ_	−0.37 **		
	Altitude	0.51 **		
**CWE_END_**	AIT	0.1 **	32.7	6262
	TPI	−0.06 **		
	pH	0.09 **		
	Altitude	0.39 **		
	CS	−0.09 **		
**PE**	Geology	0.25 *	26.0	21959
	PET_DRQ_	−0.55 *		
**RPE**	AIT	−0.14 **	28.5	24798
	TPI	−0.18 **		
	PET_DRQ_	−0.37 **		
	Altitude	0.51 **		

## Data Availability

Not applicable.

## Supplementary Materials

The following are available online at https://www.mdpi.com/2079-7737/10/2/72/s1, Extended Materials and Methods, Figure S1: Red colouring indicates cells with Level-2 (top 5%) CWE_END_ values. Blue colouring indicates cells with values from the 5% percentile (CWE_END_ coldspots). Black colouring indicates the areas where ultramafic rocks occur in Greece, Figure S2: Red colouring indicates cells with Level-3 (top 10%) CWE_END_ values. Blue colouring indicates cells with values from the 10% percentile (CWE_END_ coldspots). Black colouring indicates the areas where ultramafic rocks occur in Greece, Figure S3: Randomization results (999 runs) for the CWE_END_ metric. Pink and blue colouring indicates areas with statistically significantly lower and higher than expected corrected weighted endemic richness. NS: non-significant, Figure S4: Randomization results (999 runs) for the CWE_NAT_ metric. Pink and blue colouring indicates areas with statistically significantly lower and higher than expected corrected weighted endemic richness. NS: non-significant, Figure S5: Map of significant phylogenetic endemism (PE) identified by the categorical analysis of neo- and paleo-endemism (CANAPE) analysis for the endemic plant taxa occurring in Greece. Dark green colouring indicates the areas where ultramafic rocks occur in Greece. Dark blue colouring indicates the climatically stable areas for the past 4 My, Figure S6: Map of significant phylogenetic endemism (PE) identified by the categorical analysis of neo- and paleo-endemism (CANAPE) analysis for the endemic plant taxa occurring in Greece. Light brown polygons depict the Special Areas of Conservation of the Natura 2000 network in Greece, Figure S7: Red colouring indicates cells identified as Priority Hotspots with Level-1 (top 1%) CWE_END_ and PE values that are inside the Special Areas of Conservation of the Natura 2000 network. Blue colouring indicates cells identified as Priority Hotspots with Level-1 (top 1%) CWE_END_ and PE values that are outside the Special Areas of Conservation of the Natura 2000 network. Green polygons depict the Special Areas of Conservation of the Natura 2000 network in Greece, Figure S8: Red colouring indicates cells identified as Priority Hotspots with Level-2 (top 5%) CWE_END_ and PE values that are inside the Special Areas of Conservation of the Natura 2000 network. Blue colouring indicates cells identified as Priority Hotspots with Level-2 (top 5%) CWE_END_ and PE values that are outside the Special Areas of Conservation of the Natura 2000 network. Green polygons depict the Special Areas of Conservation of the Natura 2000 network in Greece, Table S1: Summary statistics regarding plant species richness for every type of endemism centre identified by the Categorical Analysis of Neo- and Paleo-Endemism (CANAPE) analysis in Greece. SR: species richness. SD: standard deviation. Unique SR refers to the taxa that occur exclusively in a single type of endemism centre, Table S2: Summary statistics regarding altitude (m a.s.l.), climate stability and the global human modification index for the different types of endemism centres, as well as for the not-significant sites. NS: not-significant. SD: standard deviation. CS: climate stability. GHM: Global Human Modification index. * denotes a *p*-value < 0.001 in the Kruskal-Wallis ANOVA, Table S3: Percent overlap (%) between the Special Areas of Conservation of the Natura 2000 network in Greece and the endemism centres detected by the Categorical Analyses of Neo- and Paleo-Endemism (CANAPE). The extent (in km2) of each CANAPE category is also presented, Table S4: Percent overlap (%) between the Special Areas of Conservation of the Natura 2000 network in Greece and: (i) the Corrected Weighted Endemism hotspots for the Greek endemic plant taxa (CWE_END_) and (ii) the Priority Hotspots detected by our analyses. PE: Phylogenetic endemism. L1, L2 and L3 refer to the 99%, 95% and 90% percentile, respectively. The Priority Hotspots are defined here as any cells belonging to the 1%, 5% and 10% of cells that had the highest score for both the CWE_END_ and PE indices, the two geographically-weighted variants of taxonomic and phylogenetic species richness, respectively.
