# Supplementary material for: Plant Endemism Centres and Biodiversity Hotspots in Greece
Source: Biology (Basel). 2021 Jan 20;10(2):72. doi: 10.3390/biology10020072 (PMC7909545; doi:10.3390/biology10020072)
Supplement: Supplementary file 1 [file biology-10-00072-s001.pdf]

Plant endemism centres and biodiversity hotspots in Greece

Biology

Extended Materials and Methods

1. Phylogenetic diversity

We estimated for each grid cell the phylogenetic alpha diversity (PD – sensu [1]) of the species inhabiting each of the grid cells with the 'picante' 1.6-2 R package [2] and the standardized effect size scores with 'PhyloMeasures' 2.1 R package [3]. We tested for non-random patterns in PD by estimating their standardized effect size (SES) scores as

$$\text{SES} = \frac{X_{obs} - mean(X_{null})}{s.d.(X_{null})} \qquad \text{Eq. (1)}$$

where X_{obs} is the observed score within each grid cell and *mean* (X_{NULL}) and *standard deviation* (X_{NULL}) are the mean and standard deviation of a null distribution of scores generated by shuffling taxa labels of the grid cell-by-species matrix 999 times. We assessed the statistical significance of the SES scores by calculating two-tailed p-values (quantiles) as:

$$\text{p-values} = \frac{rank_{obs}}{runs+1} \qquad \text{Eq. (2)}$$

where $rank_{obs}$ is the rank of the observed scores compared with those of their null distributions, and *runs* is the number of randomizations [2,3]. SES scores with $p < 0.05$ and $p > 0.95$ were considered as significantly lower and higher than expected for a given PD value, respectively. Positive SES values indicate phylogenetic overdispersion, whereas negative SES values indicate phylogenetic clustering. The greater sensitivity of SES_{PD} to more terminal structure makes it better suited to explore assembly processes working at finer temporal and spatial scales [4].

2. Biodiversity analyses

We followed the CANAPE protocol for spatial phylogenetics analyses as set out in [5,6]. We carried out all the relevant analyses in Biodiverse version 3.0 [5]. We first calculated phylogenetic endemism (PE – [7]) and relative phylogenetic endemism (RPE – [6]). PE is the total branch length from the dated phylogenetic tree of the lineages present at a grid cell divided by the range sizes of the respective lineages. RPE is the ratio between PE measured from the original phylogeny in relation to the PE estimated from a phylogeny with equally distributed branch lengths (see [6] for more details). Relative phylogenetic diversity (RPD) is also a ratio that compares the phylogenetic diversity (PD) observed on the actual tree in the numerator to that observed on a comparison tree in the denominator. To make them easily comparable between analyses, the trees in both the numerator and the denominator are scaled such that branch lengths are calculated as a fraction of the total tree length. The comparison tree retains the actual tree topology but makes all branches of equal length. Thus, RPD is PD measured on the actual tree divided by PD measured on the comparison tree, while RPE is PE measured on the actual tree divided by PE measured on the comparison tree [6]. RPE is the basis for the Categorical Analyses of Neo- and Paleo-Endemism (CANAPE).

2.1 Randomization tests

We assessed the statistical significance of PD, PE, RPD and RPE by following [6] approach. We compared the actual PE and RPE values of each grid cells to the 999 values of a null distribution, using the 'rand_structured' option in Biodiverse. In this model, species occurrences in grid cells are randomly reassigned to grid cells without replacement, thus keeping constant both the total number of grid cells for each species and the SR of each grid cell. We ran 999 randomisations, calculating PD, PE, RPD and RPE for each run. These values formed a null distribution for each grid cell for use in non-parametric tests of the significance of observed values. We estimated p-values from a two-tailed distribution of values to identify areas with higher (> 0.975) or lower (< 0.025) PE or RPE than the null distribution [6].

2.2 CANAPE

CANAPE is a two-step procedure discriminating grid cells with significantly high PE in neo- or paleo-endemism based on species occurrences and the dated phylogenetic tree [6].

First, to determine whether a site is a centre of significantly high endemism, a grid cell needs to be significantly high (one-tailed test, $\alpha = 0.05$) in the numerator of RPE, the denominator or both.

If (and only if) grid cells pass one of those tests, then they are divided into four meaningful, non-overlapping categories of centres of endemism [6]. If a point is significantly high in the RPE ratio (two-tailed test, $\alpha = 0.05$), then it is a centre of paleo-endemism (contains significantly more endemic species on long branches). If a point is significantly low in the RPE ratio (two-tailed test, $\alpha = 0.05$), then it is a centre of neo-endemism (contains significantly more endemic species on short branches). If it is significantly high in both the numerator and the denominator (taken alone), but not significant for RPE, then it is a centre of mixed endemism. Mixed endemism can be interpreted as a centre of endemism having a mix of rare long and rare short branches, so not significantly dominated by either paleo-endemism or neo-endemism. The mixed endemism areas are further subdivided: those grid cells that are significantly high in both the numerator and the denominator at the $\alpha = 0.01$ level are termed super-endemic sites (i.e., highly significant concentration of endemic long and short branches – [6]).

All analyses were performed using Perl wrapper functions to run Biodiverse in R modified from https://github.com/NunzioKnerr/biodiverse_pipeline.

4. References

1. Faith, D.P. Conservation evaluation and phylogenetic diversity. *Biol. Conserv.* **1992**, doi:10.1016/0006-3207(92)91201-3.
2. Kembel, S.W.; Cowan, P.D.; Helmus, M.R.; Cornwell, W.K.; Morlon, H.; Ackerly, D.D.; Blomberg, S.P.; Webb, C.O. Picante: R tools for integrating phylogenies and ecology. *Bioinformatics* **2010**, doi:10.1093/bioinformatics/btq166.
3. Tsirogiannis, C.; Sandel, B. PhyloMeasures: a package for computing phylogenetic biodiversity measures and their statistical moments. *Ecography.* **2016**, *39*, 709–714, doi:10.1111/ecog.01814.
4. Mazel, F.; Renaud, J.; Guilhaumon, F.; Mouillot, D.; Gravel, D.; Thuiller, W. Mammalian phylogenetic diversity-area relationships at a continental scale. *Ecology* **2015**, doi:10.1890/14-1858.1.
5. Laffan, S.W.; Lubarsky, E.; Rosauer, D.F. Biodiverse, a tool for the spatial analysis of biological and related diversity. *Ecography.* **2010**, *33*, 643–647, doi:10.1111/j.1600-0587.2010.06237.x.
6. Mishler, B.D.; Knerr, N.; González-Orozco, C.E.; Thornhill, A.H.; Laffan, S.W.; Miller, J.T. Phylogenetic measures of biodiversity and neo-and paleo-endemism in Australian acacia. *Nat. Commun.* **2014**, *5*, doi:10.1038/ncomms5473.
7. Rosauer, D.; Laffan, S.W.; Crisp, M.D.; Donnellan, S.C.; Cook, L.G. Phylogenetic endemism: A new approach for identifying geographical concentrations of evolutionary history. *Mol. Ecol.* **2009**, doi:10.1111/j.1365-294X.2009.04311.x.

Supplementary tables and figures

Supplementary tables

Table S1. Summary statistics regarding plant species richness for every type of endemism centres identified by the Categorical Analysis of Neo- and Paleo-Endemism (CANAPE) analysis in Greece. SR: species richness. SD: standard deviation. Unique SR refers to the taxa that occur exclusively in a single type of endemism centre.

Type	Unique SR	Median SR	Min SR	Max SR	SD SR
**Mixed**	144	29	1	99	23.9
**Neo**	32	9.5	1	36	9.8
**NS**	267	37.5	1	116	25.9
**Paleo**	11	16.5	1	104	25.7
**Super**	23	11	2	40	11.7

Table S2. Summary statistics regarding altitude (m a.s.l.), climate stability and the global human modification index for the different types of endemism centres, as well as for the not-significant sites. NS: not-significant. SD: standard deviation. CS: climate stability. GHM: Global Human Modification index. * denotes a p-value < 0.001 in the Kruskal-Wallis ANOVA.

Metric	Variable	Mixed	Neo	NS	Palaeo	Super
Median	Altitude	647	375	336	736	825
Min	Altitude	0	0	0	19	90
Max	Altitude	2121	1841	1871	2120	2286
SD	Altitude	547	428	397	598	818
Median	Climate stability	0.17	0.22	0.11	0.12	0.19
Min	Climate stability	0.02	0.02	0.01	0.06	0.11
Max	Climate stability	0.50	0.46	0.67	0.39	0.40
SD	Climate stability	0.11	0.13	0.08	0.08	0.10
Median	GHM	0.31	0.40	0.38	0.28	0.23
Min	GHM	0.06	0.11	0.06	0.13	0.11
Max	GHM	0.87	0.79	0.93	0.75	0.44
SD	GHM	0.16	0.16	0.15	0.13	0.09
Median	Longitude	22.90	23.60	22.70	22.30	22.90
Min	Longitude	19.90	20.10	19.40	20.80	20.80
Max	Longitude	28.20	26.90	28.20	25.90	29.60
SD	Longitude	1.77	1.60	1.71	1.27	2.36
Median	Latitude	39.60	40.40	38.10	38.10	40.10
Min	Latitude	35.10	36.50	34.80	35.10	36.10
Max	Latitude	41.50	41.50	41.60	40.90	40.50
SD	Latitude	1.68	1.52	1.64	1.77	1.40

Regarding elevation, neo-endemism centres were statistically significantly different from the other types of endemism centres.

Regarding climate stability, paleo-endemism centres were statistically significantly different from the other types of endemism centres.

Regarding the global human modification index, all combinations were statistically significantly different, except for the mixed-paleo combination.

Regarding longitude and latitude, the only combination that was statistically significantly different for both variables, was the paleo- and neo-endemism centre combination.

Table S3. Percent overlap (%) between the Special Areas of Conservation of the Natura 2000 network in Greece and the endemism centres detected by the Categorical Analyses of Neo- and Paleo-Endemism (CANAPE). The extent (in km^2) of each CANAPE category is also presented.

Type	Mixed	Neo	NS	Paleo	Super
**% PA overlap**	57.8	45.8	30	38.9	84.5
**% Conservation gaps**	42.2	54.2	70	61.1	15.5
**Extent**	2898	673	16687	355	182

Table S5. Percent overlap (%) between the Special Areas of Conservation of the Natura 2000 network in Greece and: i) the Corrected Weighted Endemism hotspots for the Greek endemic plant taxa (CWE_{END}) and ii) the Priority Hotspots detected by our analyses. PE: Phylogenetic endemism. L1, L2 and L3 refer to the 99%, 95% and 90% quantile, respectively. The Priority Hotspots are defined here as any cells belonging to the 1%, 5% and 10% of cells that had the highest score for both the CWE_{END} and PE indices, the two geographically-weighted variants of taxonomic and phylogenetic species richness, respectively.

Type	Level	% Overlap	% Conservation gaps
Priority Hotspots (PE – CWE_{END})	L1	96.7	3.3
	L2	81.7	18.3
	L3	74.9	25.1
CWE_{END}	L1	95.7	4.3
	L2	78.8	21.2
	L3	72.2	27.8

Supplementary Figures

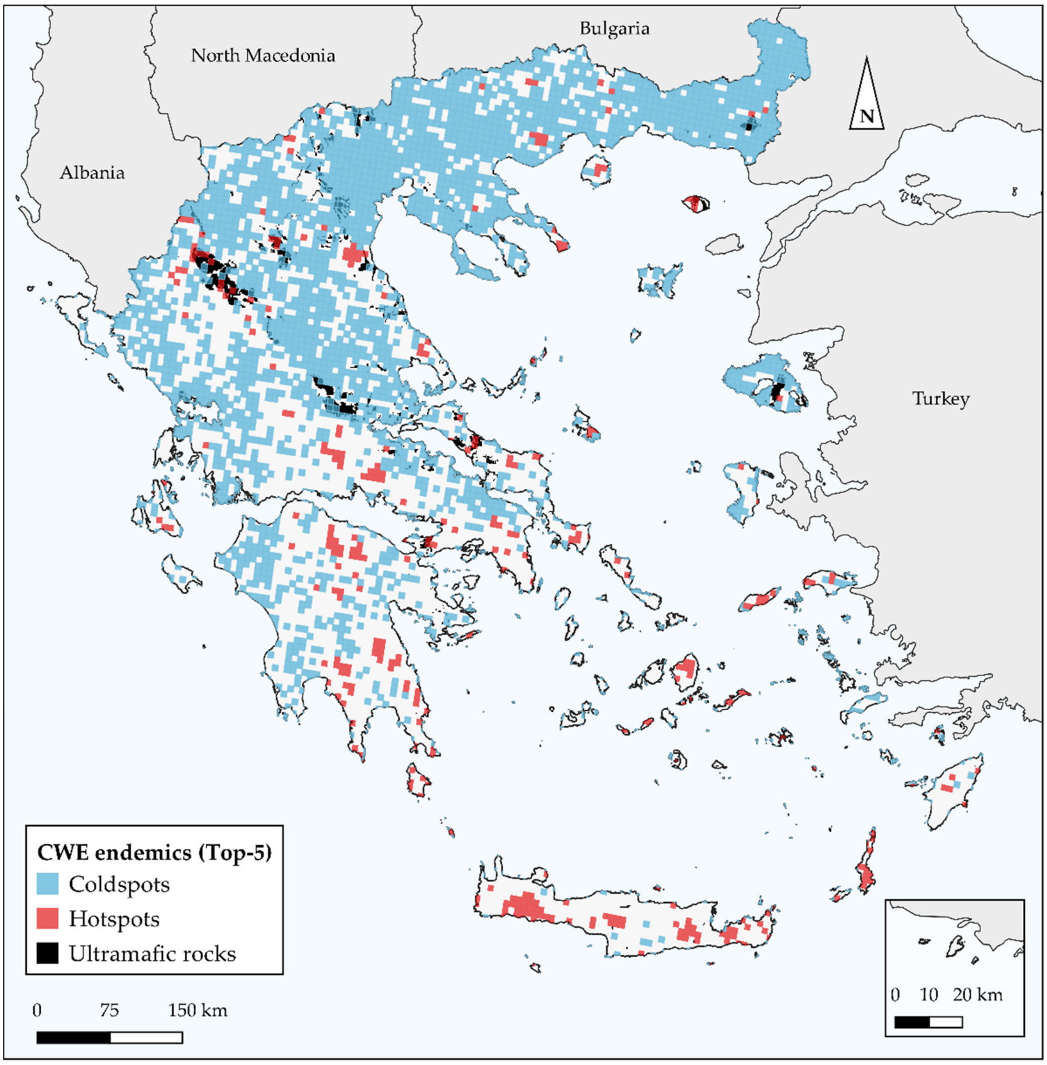

Figure S1. Red colouring indicates cells with Level-2 (top-5%) CWE_{END} values. Blue colouring indicates cells with values from the 5% percentile (CWE_{END} coldspots). Black colouring indicates the areas where ultramafic rocks occur in Greece.

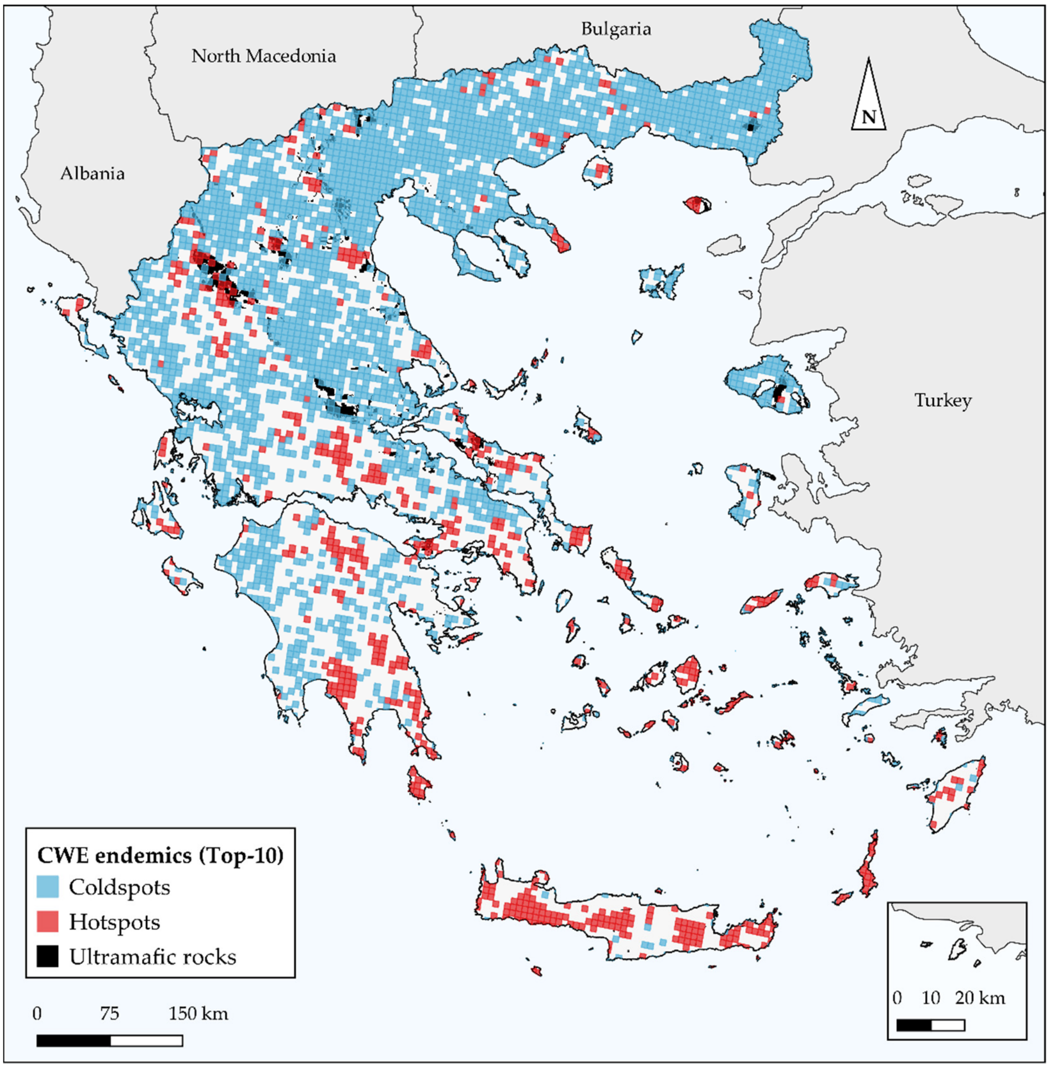

Figure S2. Red colouring indicates cells with Level-3 (top-10%) CWE_{END} values. Blue colouring indicates cells with values from the 10% percentile (CWE_{END} coldspots). Black colouring indicates the areas where ultramafic rocks occur in Greece.

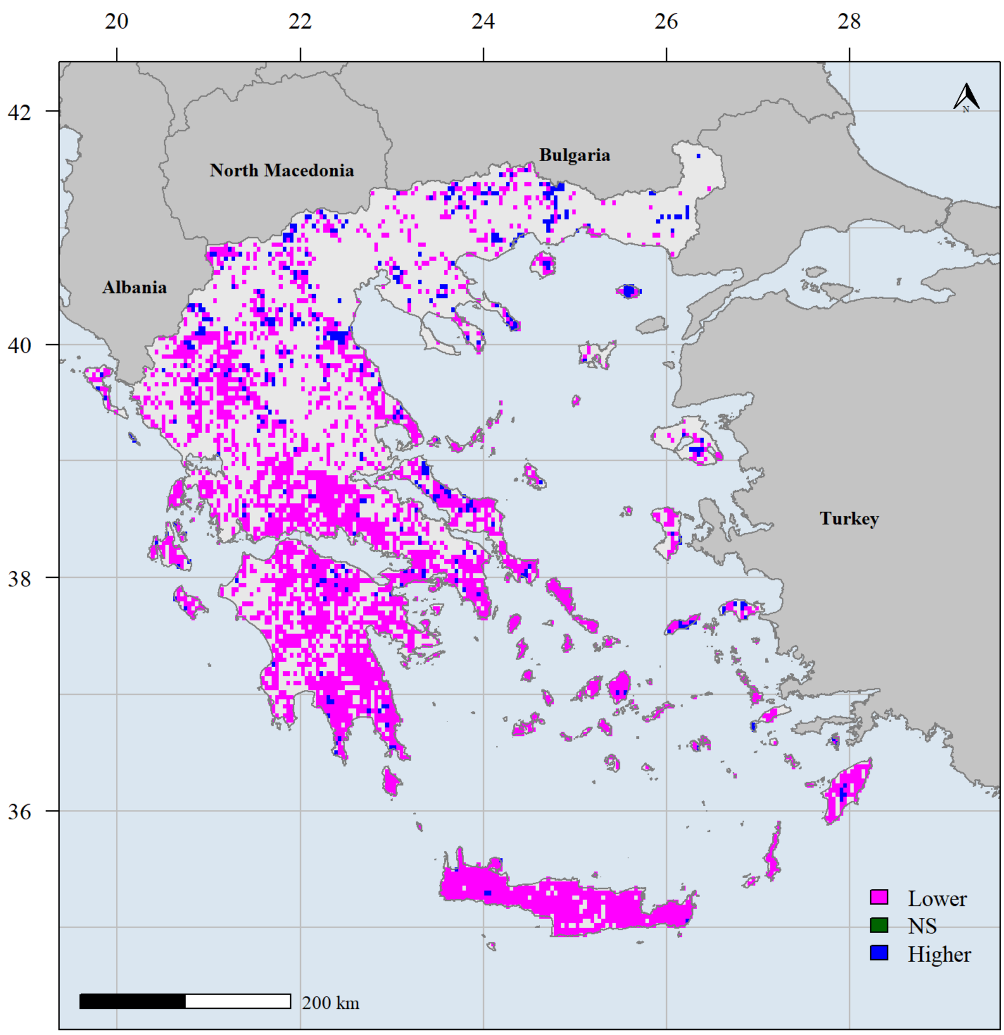

Figure S3. Randomization results (999 runs) for the CWE_{END} metric. Pink and blue colouring indicates areas with statistically significantly lower and higher than expected corrected weighted endemic richness. NS: non-significant.

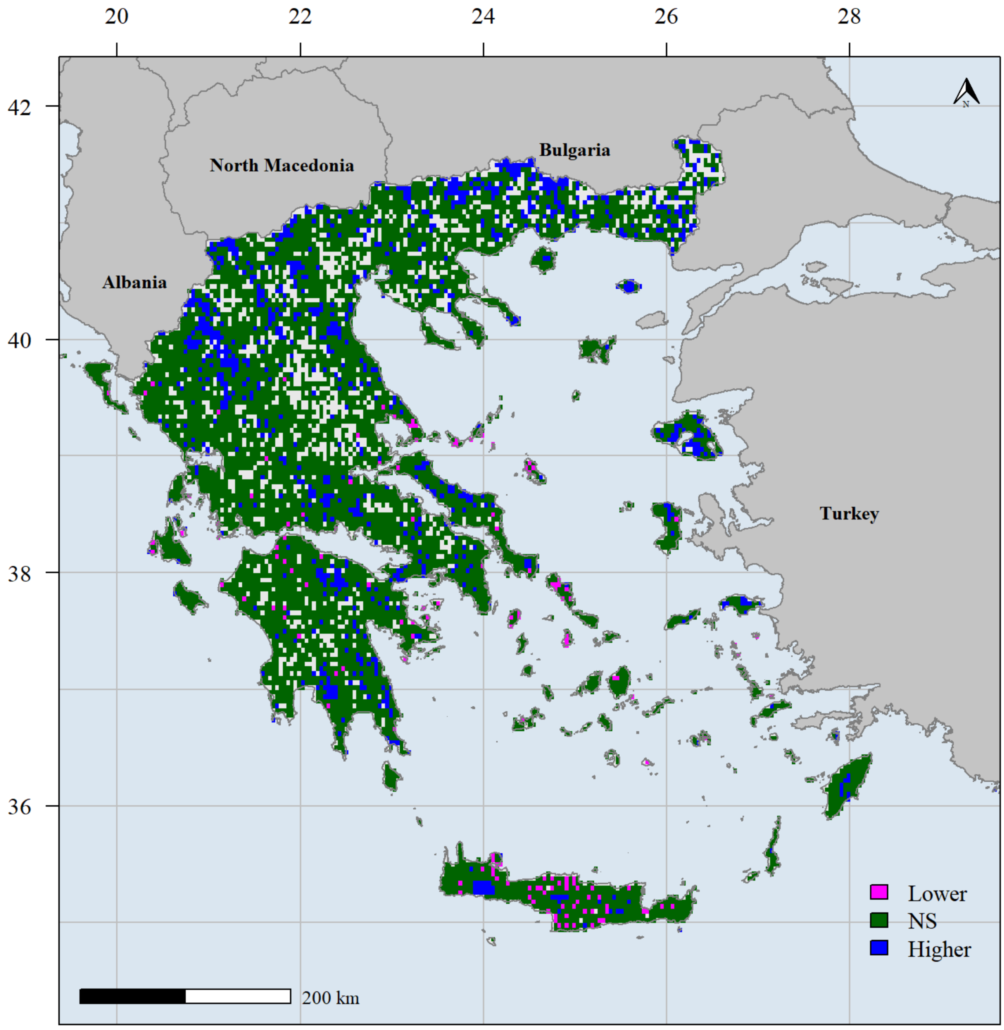

Figure S4. Randomization results (999 runs) for the CWEnat metric. Pink and blue colouring indicates areas with statistically significantly lower and higher than expected corrected weighted endemic richness. NS: non-significant.

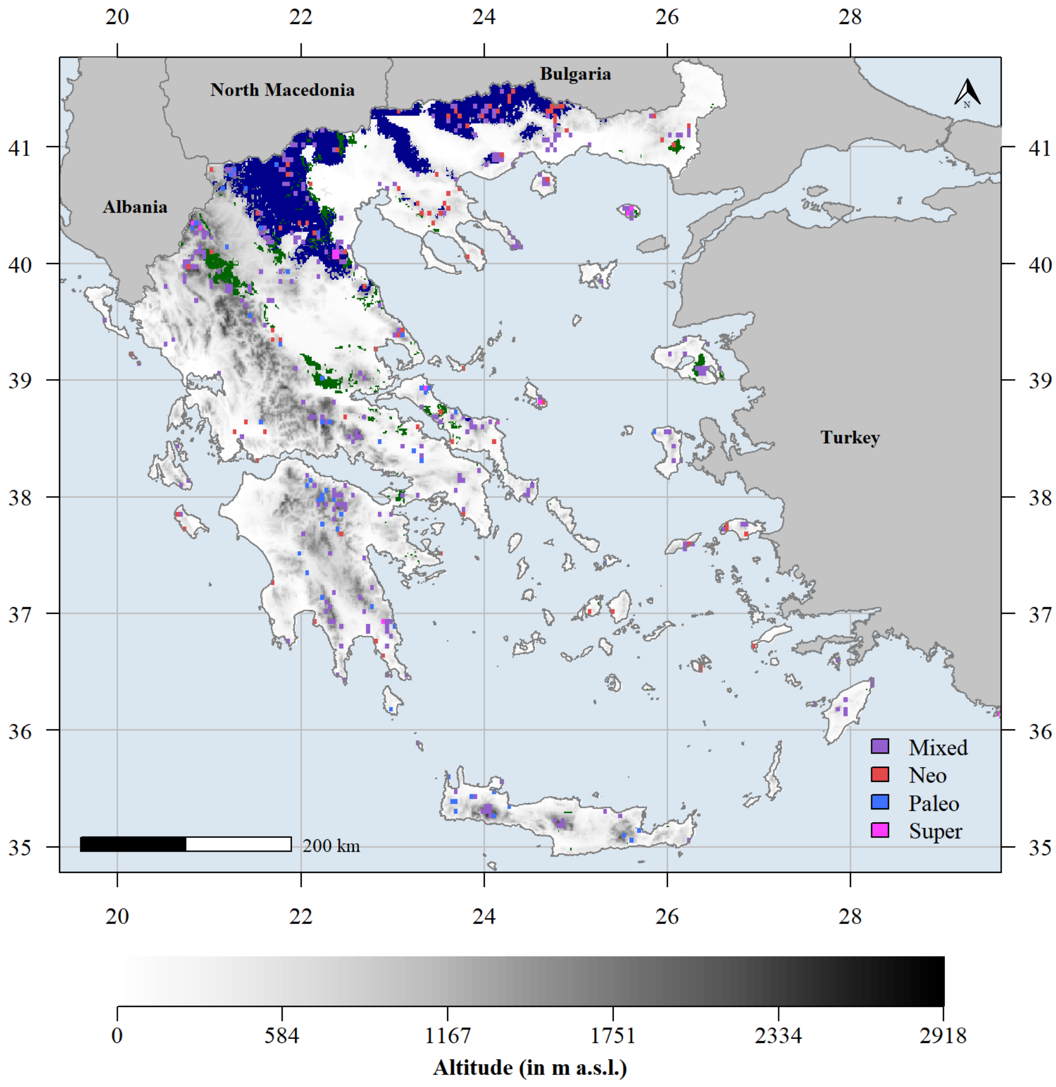

Figure S5. Map of significant phylogenetic endemism (PE) identified by the categorical analysis of neo- and paleo-endemism (CANAPE) analysis for the endemic plant taxa occurring in Greece. Dark green colouring indicates the areas where ultramafic rocks occur in Greece. Dark blue colouring indicates the climatically stable areas for the past 4 My.

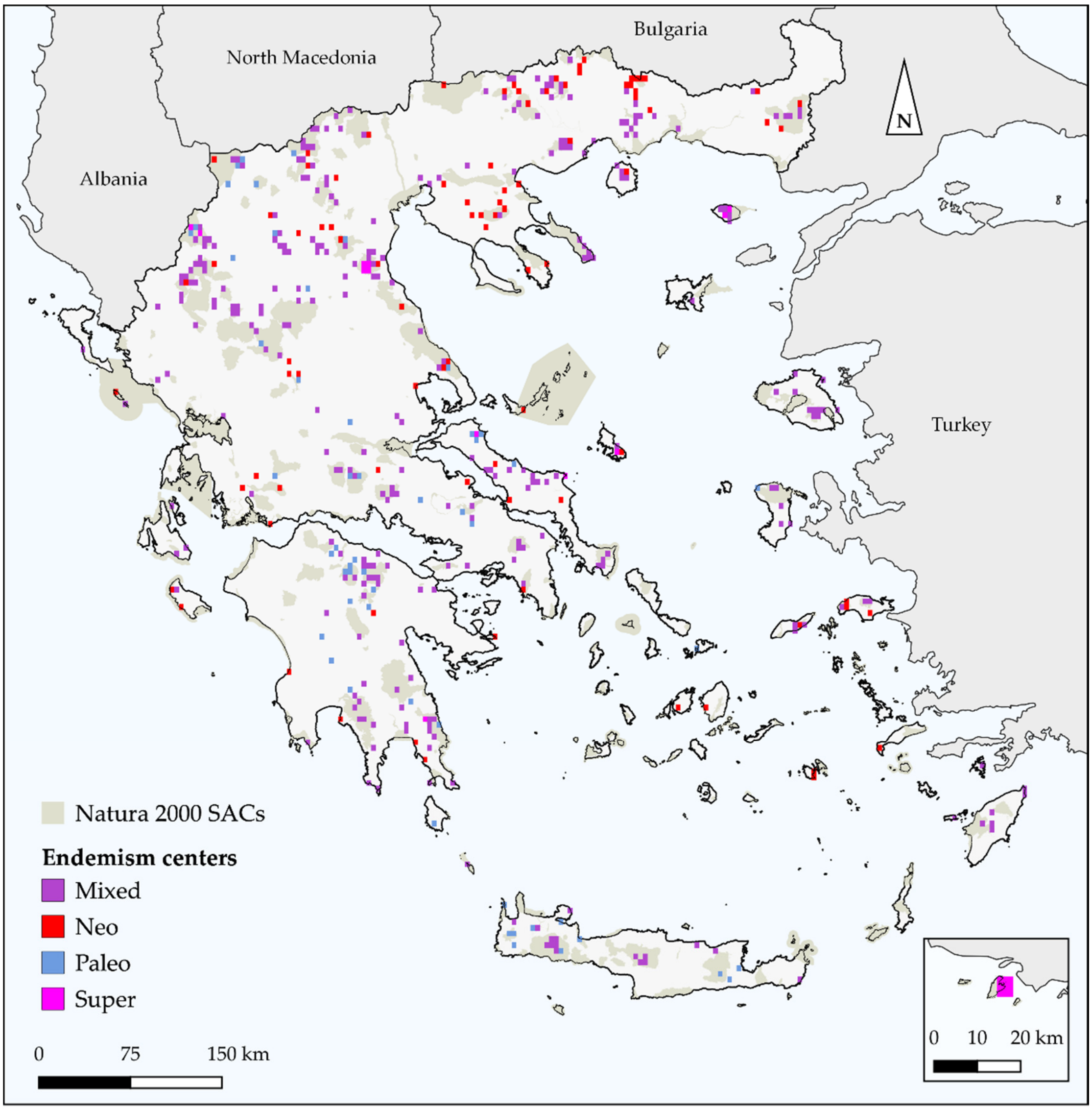

Figure S6. Map of significant phylogenetic endemism (PE) identified by the categorical analysis of neo- and paleo-endemism (CANAPE) analysis for the endemic plant taxa occurring in Greece. Light brown polygons depict the Special Areas of Conservation of the Natura 2000 network in Greece.

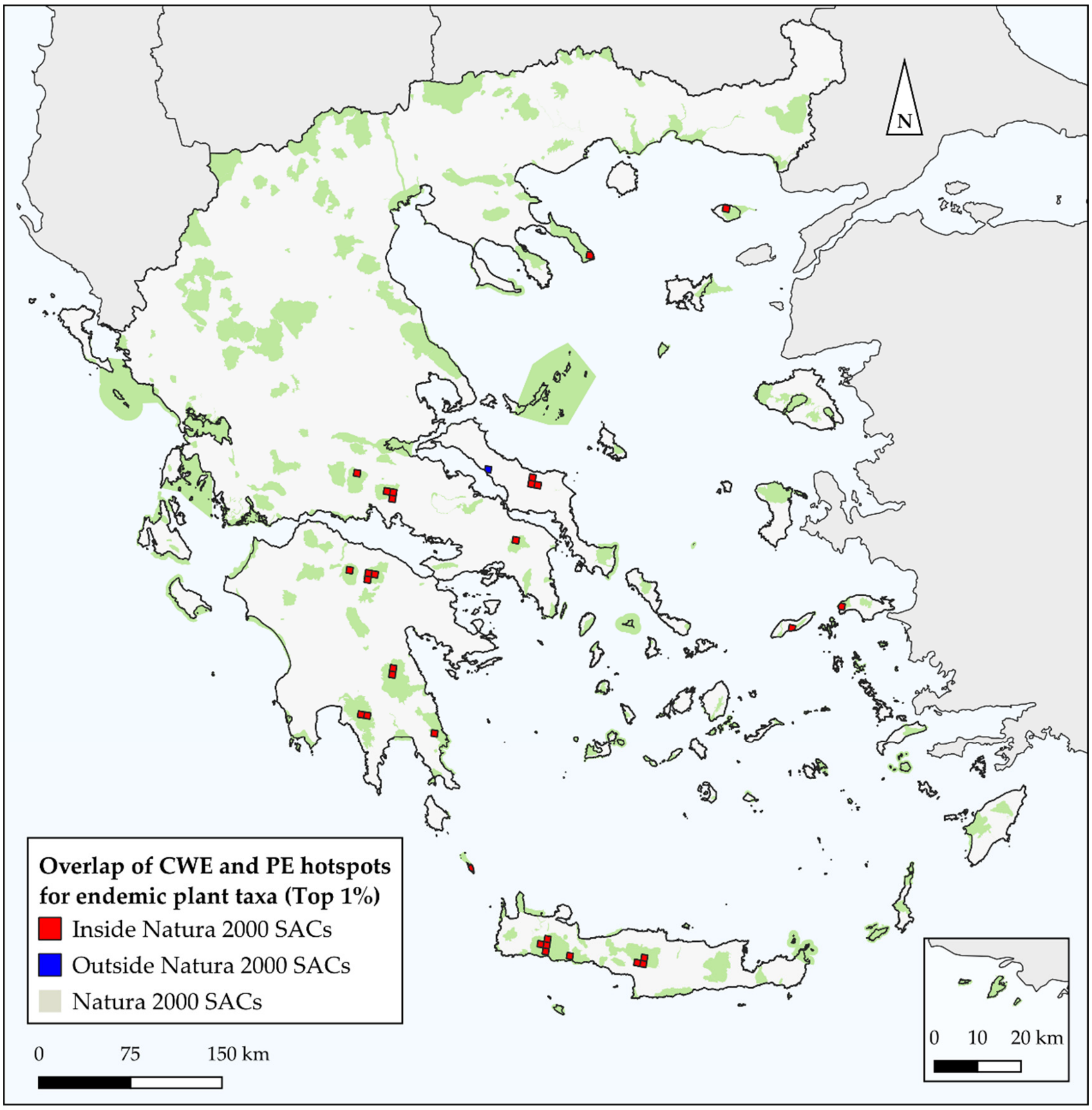

Figure S7. Red colouring indicates cells identified as Priority Hotspots with Level-1 (top 1%) CWEEND and PE values that are inside the Special Areas of Conservation of the Natura 2000 network. Blue colouring indicates cells identified as Priority Hotspots with Level-1 (top 1%) CWEEND and PE values that are outside the Special Areas of Conservation of the Natura 2000 network. Green polygons depict the Special Areas of Conservation of the Natura 2000 network in Greece.

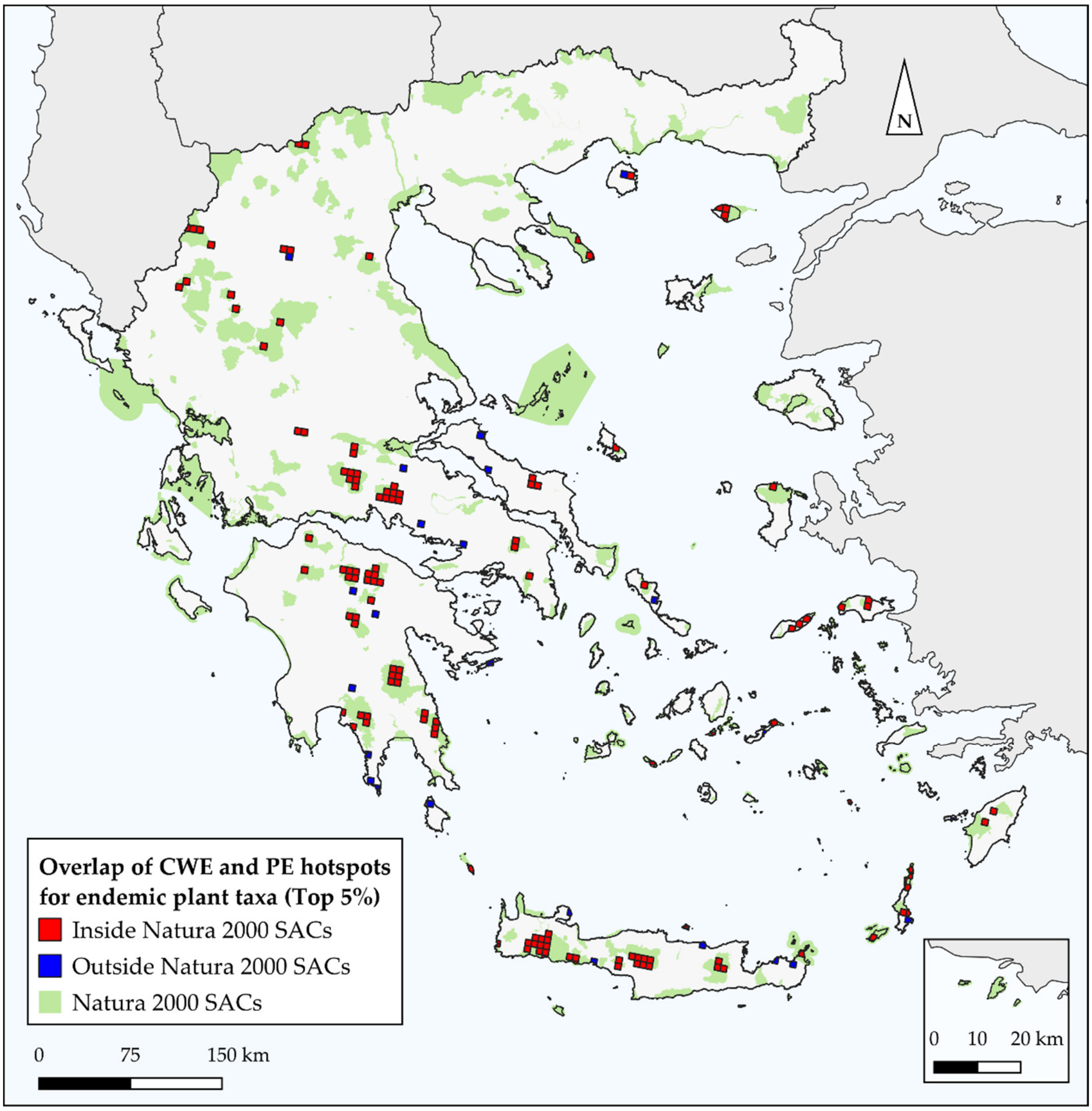

Figure S8. Red colouring indicates cells identified as Priority Hotspots with Level-2 (top 5%) CWE_{END} and PE values that are inside the Special Areas of Conservation of the Natura 2000 network. Blue colouring indicates cells identified as Priority Hotspots with Level-2 (top 5%) CWE_{END} and PE values that are outside the Special Areas of Conservation of the Natura 2000 network. Green polygons depict the Special Areas of Conservation of the Natura 2000 network in Greece.
